# Global trends and research profile of antibiotic heteroresistance: a bibliometric and visual analysis

**DOI:** 10.3389/fmicb.2026.1723669

**Published:** 2026-07-17

**Authors:** Jie Hou, Chen Peng, Weilin Chen, Liang Peng, Ying Ma, Mei Kang

**Affiliations:** 1 Department of Laboratory Medicine, West China Tianfu Hospital, Sichuan University, Chengdu, China; 2 Department of Laboratory Medicine, West China Hospital, Sichuan University, Chengdu, China; 3 Sichuan Clinical Research Center for Laboratory Medicine, Chengdu, China; 4 Laboratory Medicine Research Center of West China Hospital, Sichuan University, Chengdu, China; 5 Health Management Center, West China Hospital, Sichuan University, Chengdu, China

**Keywords:** antibiotic heteroresistance, antifungal heteroresistance, antimicrobial heteroresistance, antimicrobial resistance, bibliometric analysis

## Abstract

**Introduction:**

Antibiotic heteroresistance challenges clinical treatment, risking adverse outcomes and antimicrobial resistance emergence. This study aims to explore its research status and trends.

**Methods:**

We screened global publications on antibiotic heteroresistance from the Web of Science Core Collection, Scopus and PubMed databases. Bibliometrix and VOSviewer were used to conduct the bibliometric and visual analysis of the publications.

**Results:**

In total, 1516 publications on antibiotic heteroresistance were included. The number of publications and citations increased with time, most notably in the last two decades. Among the 377 sources, the most active and most cited journal was *Antimicrobial Agents and Chemotherapy*. The most productive and most cited author were Michael J. Rybak and Keiichi Hiramatsu, respectively.Emory University and Monash University were the institutions with the most publications and citations, respectively. The USA had the highest publications and citations. Emory University and Monash University were the institutions with the most publications and citations, respectively. The USA had the highest publications and citations. A total of 1758 author keywords and five main clusters were identified, including heteroresistance terms cluster, Gram-positive bacteria cluster, Gram-negative bacteria cluster, *Mycobacterium tuberculosis* cluster and antifungal heteroresistance cluster. The top ten antibiotics and microorganisms with keywords co-occurrence frequency were colistin, vancomycin, polymyxins, daptomycin, rifampin, fluconazole, fluoroquinolones, linezolid, tigecycline, clarithromycin, MRSA, *Staphylococcus aureus, hVISA, Acinetobacter baumannii, Mycobacterium tuberculosis, Helicobacter pylori, Klebsiella pneumoniae*, VISA, *Pseudomonas aeruginosa, Staphylococcus*.

**Conclusion:**

Findings reveal global trends and research profiles of antibiotic heteroresistance. Useful for researchers, microbiologists, and clinicians to understand the field and guide future studies.

## 1 Introduction

Antibiotic heteroresistance is a phenotype in which a bacterial isolate contains subpopulations of cells that exhibit increased levels of antibiotic resistance compared with the main population (El-Halfawy and Valvano, 2015; Andersson et al., 2019), with highly variable prevalence reported across different bacterial species and antibiotic classes. Antibiotic heteroresistance cannot only lead to clinical treatment failure, but may also result in the widespread prevalence of new antibiotic drug resistance due to the screening of new subgroups of resistance under antibiotic pressure, exacerbating the global burden of antimicrobial resistance (AMR) (Tiseo et al., 2023). Since various Gram-negative and Gram-positive bacteria can exhibit the heteroresistance phenotype for most antibiotic classes, antibiotic heteroresistance indeed could be a long-term and seriously ignored problem (Roch et al., 2023). Accumulating evidence, including a series of well-controlled animal studies, mathematical modeling of heteroresistance, and retrospective clinical studies, indicates that heteroresistance can lead to treatment failure and is associated with persistent bacteremia, increased mortality, prolonged hospital stays, and complications (Andersson et al., 2019). Current clinical studies on heteroresistance in clinical isolates suggest that heteroresistance could be linked to clinical outcomes, including studies focusing on heteroresistance to vancomycin in *Staphylococcus aureus* (van Hal and Paterson, 2011) colistin or carbapenems heteroresistance in *Acinetobacter baumannii* (Kon et al., 2023; Fernández-Cuenca et al., 2012). However, the full landscape of microorganism-antimicrobial combinations contributing to antibiotic heteroresistance remains elusive and requires further investigation.

Although reliable methods exist for AMR detection in different microbial species, including reference standards for antimicrobial susceptibility testing of global authoritative organizations [eg., Clinical and Laboratory Standards Institute (CLSI), European Committee on Antimicrobial Susceptibility Testing (EUCAST)] and commercial assays, antimicrobial heteroresistance still lacks a unified and consistent definition across different microbial species to date (El-Halfawy and Valvano, 2015; Andersson et al., 2019; Roch et al., 2023), e.g., there are clear differences in the definition of heteroresistance across microbial species. Classically, heteroresistance in Gram-positive and Gram-negative bacteria refers to the presence of a small subpopulation (typically 1 in 10^−6^ or 10^−7^) with increased antibiotic resistance within a single clonal bacterial isolate (El-Halfawy and Valvano, 2015; Andersson et al., 2019). In contrast, descriptions of “heteroresistance” in *Mycobacterium tuberculosis* and *Helicobacter pylori* often include polyclonal coexistence of distinct strains/clones (e.g., mixed infections or within-host evolution of divergent lineages) in the same patient (polyclonal), which is conceptually distinct from the classic heteroresistance definition (El-Halfawy and Valvano, 2015; Andersson et al., 2019). Current antimicrobial heteroresistance research remains largely focused on single microbial species, making it challenging to assess the clinical significance of heteroresistance from a broader, macro-level perspective. To promote comparability of heteroresistance across studies, several key factors need to be defined and measured, including the origin and clonality, resistance level, frequency, and stability of the resistant subpopulations (Andersson et al., 2019). Besides, for a given microbial species and specific antibiotic combination, the perspective of antibiotic heterogeneous resistance research may at least cover precise definition, detection methods, microbiological characteristics, prevalence, clinical impact, pharmacokinetics/pharmacodynamics, and underlying mechanisms. With the continual expansion of antibiotic heteroresistance research in both scope and complexity, bibliometric analysis stands out as a crucial tool for the systematic evaluation of its evolving research landscape.

Bibliometric analysis is the quantitative study of published scientific literature by a set of mathematical and statistical methods to measure the quality and quantity of related literature and to study a particular research area of academic impact (for example, identification of prominent journals, core authors, institutions, countries, and influential articles), collaboration patterns, research trends, research frontiers, and hotspots (Hood and Wilson, 2001; Agarwal et al., 2016). During the last decade, numerous bibliometric studies focusing on antimicrobial resistance have been published, including research on antimicrobial resistance in *S. aureus* (Shi, 2025), *Klebsiella pneumonia* (Li Y. P. et al., 2022), *Salmonella* (Yan et al., 2024), *H. pylori* (Li et al., 2023; Yuan et al., 2023), uropathogens (Sweileh et al., 2018) and pneumonia pathogens (Ablakimova et al., 2023), as well as studies on carbapenem resistance (Sweileh et al., 2016), polymyxin resistance (Quincho-Lopez and Pacheco-Mendoza, 2021), antimicrobial resistance in the general environment (Sweileh and Mansour, 2020), natural water systems (Sahin et al., 2021), aquaculture (Xiao et al., 2023; Mohammed et al., 2025), food-producing animals (Sweileh, 2021), and wildlife (Torres et al., 2020). However, no bibliometric studies have been carried out on antibiotic heteroresistance to provide a detailed mapping of the research evolution. This gap in information hinders a clear grasp of major scientific trends in the field, the advancement of innovative methodologies, and the identification of critical gaps within the literature. Furthermore, multi-database analysis can overcome the biases and limitations associated with single-database analysis, making it more suitable for comprehensive global assessments and enhancing the ability to validate results. Web of science core collection (WOSCC) database, Scopus database, and PubMed database are the three mainstream databases in the biomedical field worldwide, known for their strict screening criteria, wide and multidimensional resource coverage, and rich clinical research. Therefore, this bibliometric study based on the three major databases aims to assess global research productivity, identify key themes of concern, ascertain emerging topics, and lay out future research directions, providing a knowledge map that delivers a profile of current antibiotic heteroresistance research.

## 2 Methods and materials

### 2.1 Study design

Following the PRISMA (Page et al., 2021) guidelines, this study used bibliometric methods, including co-authorship analysis, co-citation analysis, bibliographic coupling, and keyword analysis, to analyze publications regarding antibiotic heteroresistance in various subject categories. The aim of using the bibliometric method is to analyze the current performance and future research trends in antibiotic heteroresistance based on dimensions such as journals, authors, institutions, countries, documents, and keywords.

### 2.2 Data search and collection

**Figure 1:**
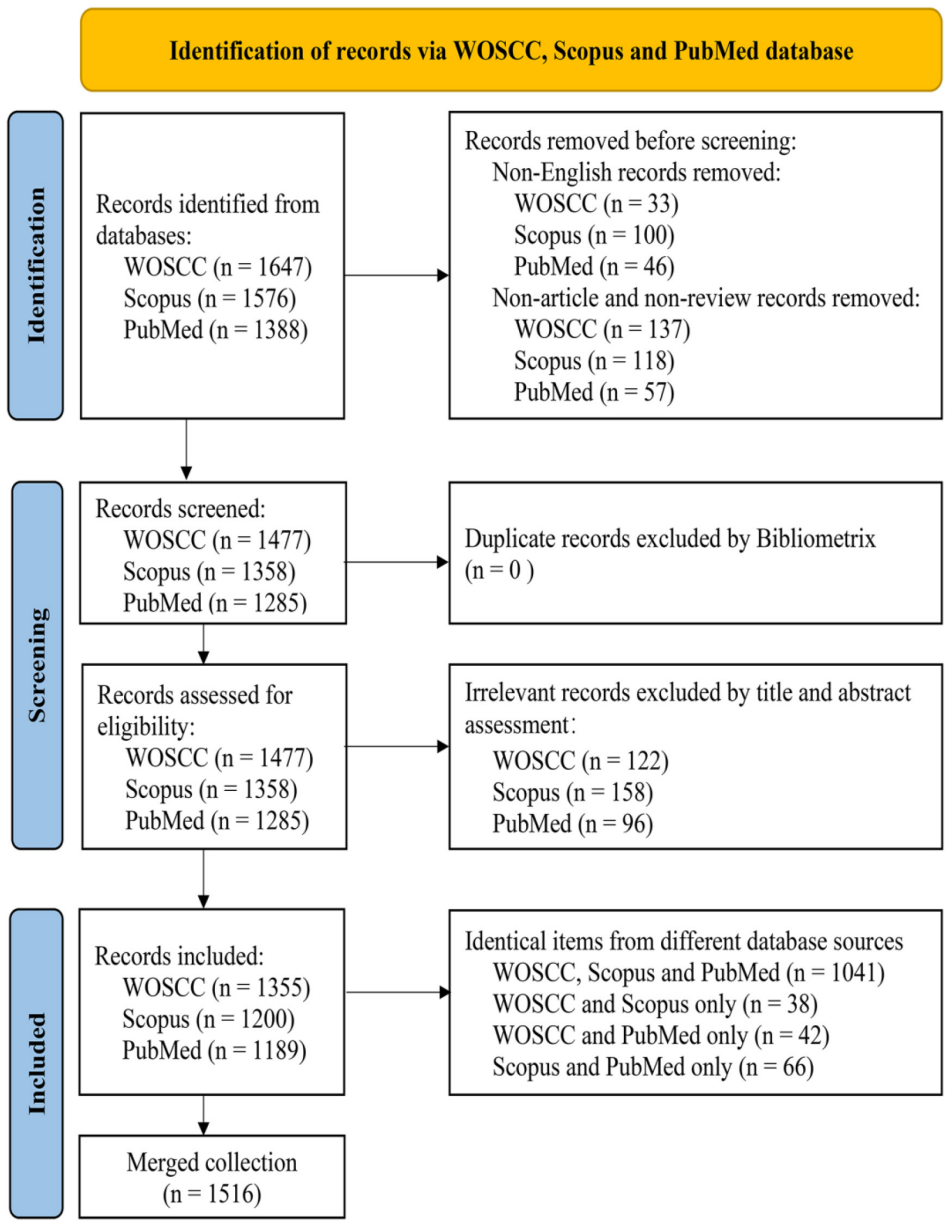
Flowchart of data filtering for the bibliometric analysis.

We focused on searching for documents in the WOSCC, Scopus, and PubMed databases, using the topic, title-abs-key, and title/abstract search strategy, respectively, and filtering by other limited items to obtain more accurate publications. The core search terms “heteroresistance” and “heterogeneous resistance”, combined with their morphological variations and truncation, were selected according to the research topics the present study focused on. The search formula for three databases was detailed in the Supplementary Tables S1–S3. Meanwhile, language was restricted to English, and document types were limited to article and review. All non-article and non-review records were excluded, including letters, meeting abstracts, editorial material, corrections, notes, news items, conference articles, short surveys, errata, editorials, comments, published errata, retracted publications, and retraction notices. In order to obtain more recent data updates, we set the cutoff date for the search for relevant items to June 30, 2025. The initially searched records were screened separately by two different investigators (J.H. and C.P.) based on title and abstract to exclude literature that was not associated with antibiotic heteroresistance. According to digital object identifier (DOI), PubMed identifier (PMID), and title plus published year, the filtered documents from different databases were deduplicated and merged into a single collection. When there were identical articles, the order of database retention for the merged collection is WOSCC, Scopus, and PubMed data, in order to ensure the standardization of records as much as possible. To maximize standardization across the records, whenever identical articles were encountered, the retention order for the merged collection was set as WOSCC, followed by Scopus, and then PubMed. In addition, the total citation counts for articles from the PubMed database were obtained from the iCite application programming interface based on their PMIDs. The final included publications represented research conducted in various subject categories involving antibiotic heteroresistance. The complete data filtering process is shown in Figure 1.

### 2.3 Bibliometric analysis

The Bibliometrix package (version 5.1.0) (Aria and Cuccurullo, 2017) was used within Rstudio (Build 563) integrated development environment, running on R (version 4.4.3), to conduct a comprehensive bibliometric analysis, especially focusing on records merge and deduplication, publication and citation profiling, journal metrics, collaboration networks among authors, institutions, and countries, as well as keyword-based assessments such as word clouds, trend topics, thematic mapping, and thematic evolution. The VOSviewer (van Eck and Waltman, 2010) (version 1.6.2) is a freely available computer program for constructing and viewing bibliometric maps, which was mainly used for co-citation analysis, bibliographic coupling, and keywords co-occurrence network. In addition, two different authors (J.H. and W.L.C.) separately completed the synonym merging table for author names, journal titles, and keywords, then formed the final synonym merging table through group discussion, and finally had the merging operation performed according to the requirements of different software.

## 3 Results

### 3.1 Publications and citations

**Figure 2:**
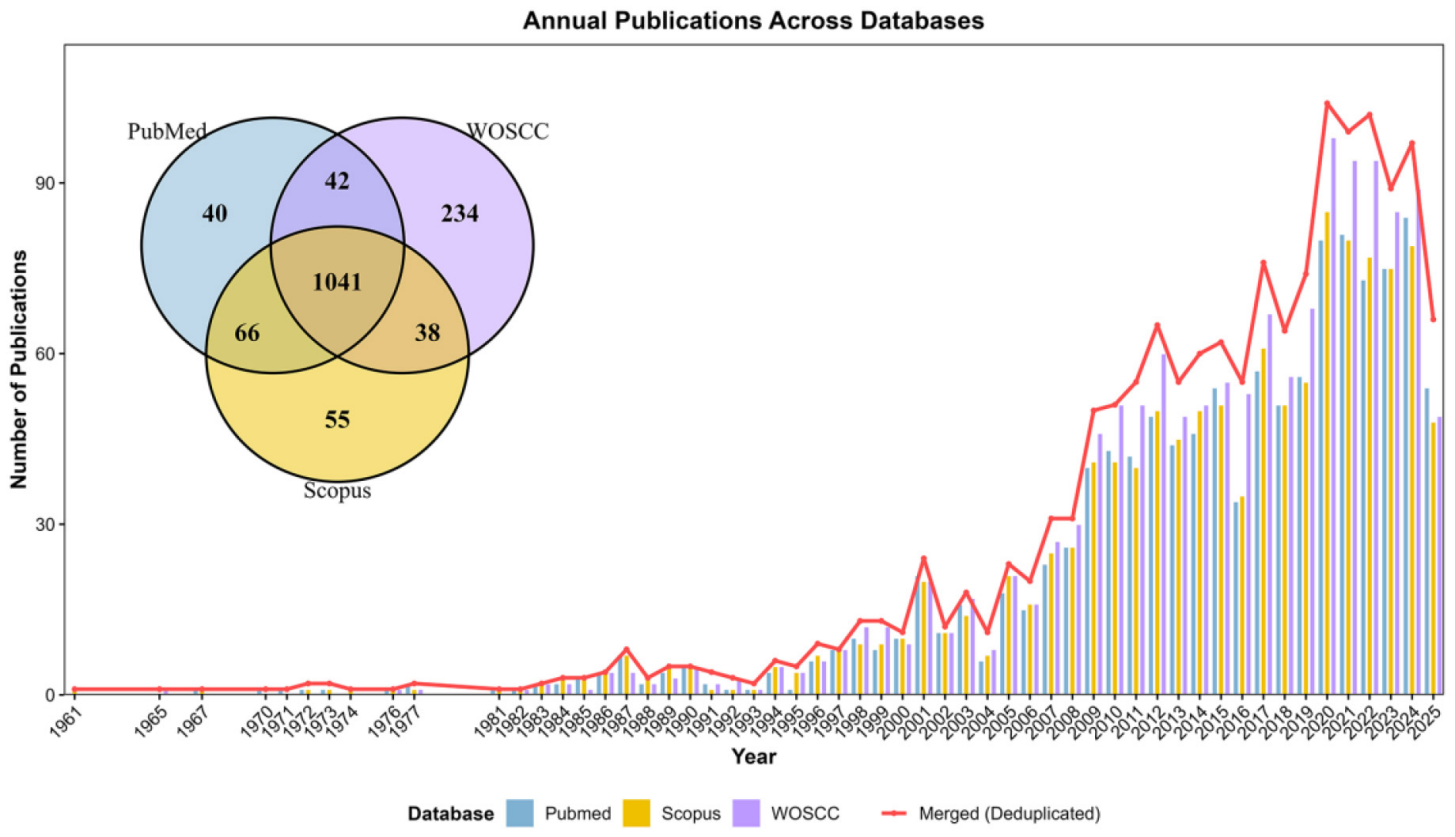
Distribution and trends of publications on antibiotic heteroresistance across multiple databases.

**Figure 3:**
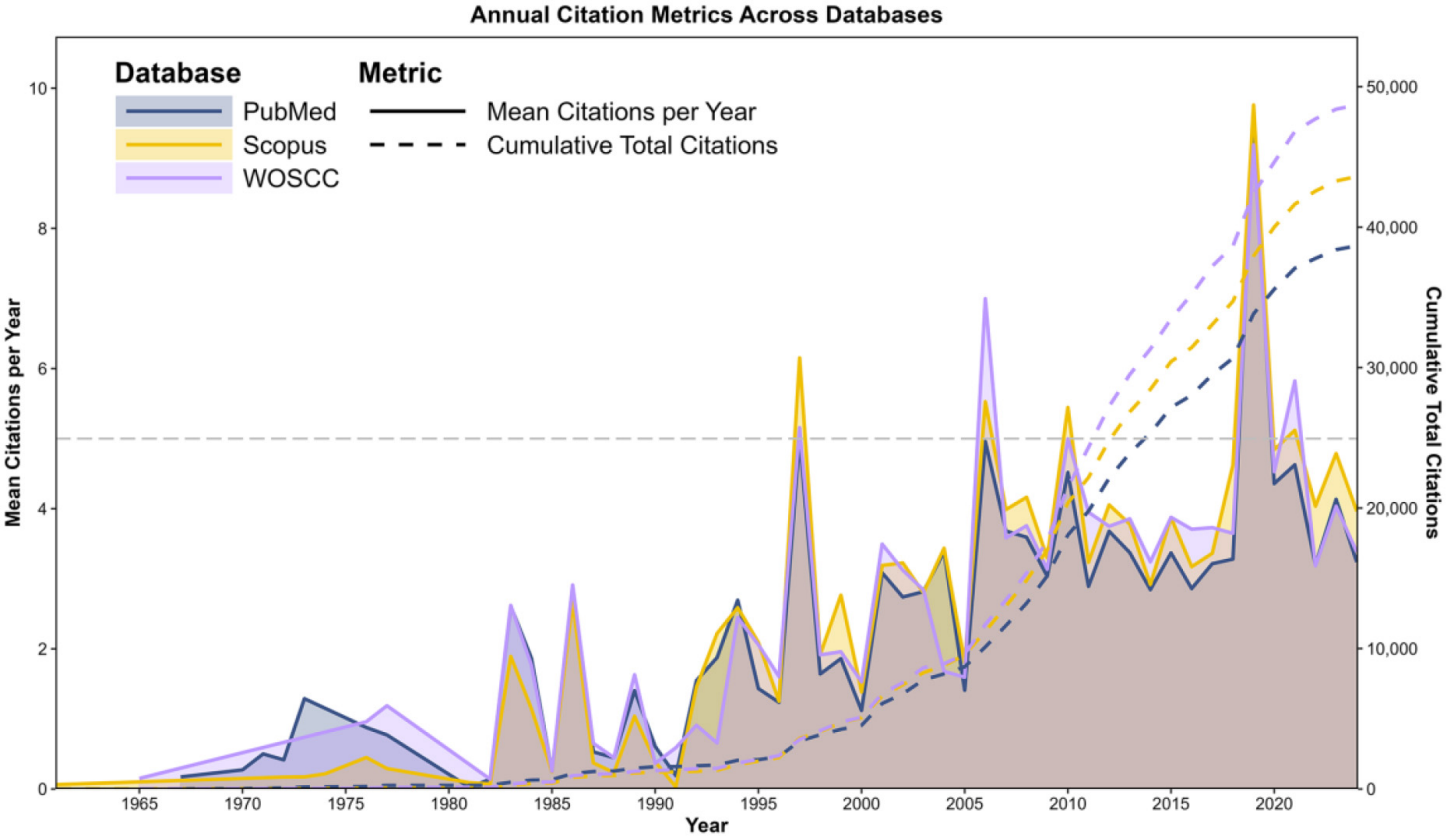
Trends of citations on antibiotic heteroresistance across multiple databases.

After searching multiple databases, a total of 1,647 publications in WOSCC, 1,576 publications in Scopus, and 1,388 publications in PubMed were initially retrieved, and 1,355 publications in WOSCC, 1,200 publications in Scopus, and 1,189 publications in PubMed were included in the final dataset after excluding ineligible records according to the exclusion criteria (Figure 1). The final included records obtained from the three databases were deduplicated and combined to form a merged collection containing 1,516 articles (Figure 1), including 1,362 articles (89.84%) and 154 reviews (10.16%). Regarding the data source distribution in the merged collection, the proportion of WOSCC was at most 89.38%, the proportion of Scopus was the second at 79.16%, the proportion of PubMed was the least at 78.43%, and the publications of the common intersection of the three databases were 1,041(68.67%) (Figure 2). Over the past few decades, there has been clear evidence showing a consistently increasing publication trend on antibiotic heteroresistance research across the three different databases (Figure 2), with an annual growth rate was 6.77% in the merged collection. The peak years with significant increases in publication numbers are 2001, 2012, and 2020, respectively (Figure 2). The changing trends of the mean citations per year, mean citations per article, and cumulative citations were very similar at the overall level across the three different databases (Figure 3 and Supplementary Figure S1). The annual average citations showed several prominent time peaks in 1997, 2006, 2010, 2019, and 2021, suggesting that these years may contain important research progress or focus (Figure 3). The annual trend of average citations per article similarly highlighted several important time points in 1997, 2006, and 2010 (Supplementary Figure S1). The cumulative citations in all three databases rose monotonically, exhibiting a pronounced acceleration post-2000 and a period of rapid growth between 2010 and 2020 (Figure 3 and Supplementary Figure S1).

### 3.2 Journal analysis

A total of 377 sources have published articles on our topic of interest, and 21 (5.57%) journals have published more than 10 items. There were 301(79.84%) journals in the WOSCC database, 309 (81.96%) journals in the Scopus database, and 288(76.39%) journals in the PubMed database. A total of 644 articles were published in the top 10 active journals, accounting for 42.48% of the total publications in the merged collection. The top six core journals, including Antimicrobial Agents and Chemotherapy, Journal of Clinical Microbiology, Journal of Antimicrobial Chemotherapy, International Journal of Antimicrobial Agents, Diagnostic Microbiology and Infectious Disease, and European Journal of Clinical Microbiology & Infectious Diseases, were identified through Bradford's Law (Table 1). Meanwhile, Antimicrobial Agents and Chemotherapy, Journal of Clinical Microbiology, and Journal of Antimicrobial Chemotherapy were the top three journals in terms of publications, total citations, average citations per item (ACI), h-index, and g-index (Table 1). The most productive and cited journal was Antimicrobial Agents and Chemotherapy, which published 172 articles (11.35%) and had the following metrics: a 2024 Journal Impact Factor (JIF) of 4.5; a 2024 CiteScore of 8.4; a h-index of 54; a g-index of 92; and an m-index of 1.00 (Table 1). Interestingly, according to the m-index ranking, the ranked top three journals were Plos One, Frontiers in Microbiology, and Scientific Reports, all of which were emerging open source journals. Journal bibliographic coupling analysis revealed strong links among the top active journals in both the WOSCC and Scopus databases (Supplementary Figure S2). Additionally, these open-access, pan-microbial journals (e.g., Frontiers in Microbiology, Microbiology Spectrum, Frontiers in Cellular and Infection Microbiology, mSphere, mBio) exhibited a more recent average publication year than those traditional subscription-based microbial journals, such as the top six core journals. Journal co-citation analysis demonstrated that the top five hub co-cited journals, including Antimicrobial Agents and Chemotherapy, Journal of Clinical Microbiology, Journal of Antimicrobial Chemotherapy, Clinical Infectious Diseases, and International Journal of Antimicrobial Agents, were shared in both WOSCC and Scopus databases based on total link strength, but with slight differences in clustering results (Supplementary Figure S2).

**Table 1:** Top 10 active journals on antibiotic heteroresistance research in the merged collection.

Journal name	Publications	Citations	ACI	JIF	CiteScore^a^	Zone^b^	h-index^c^	g-index^c^	m-index^c^
Antimicrobial Agents and Chemotherapy	172	9,763	56.76	4.5	8.4	1	54	92	1.00
Journal of Clinical Microbiology	99	4,779	48.27	5.4	12.1	1	42	66	0.98
Journal of Antimicrobial Chemotherapy	94	4,198	44.66	3.6	7.3	1	37	63	0.92
International Journal of Antimicrobial Agents	57	1,897	33.28	4.6	5.8	1	25	43	0.86
Diagnostic Microbiology and Infectious Disease	47	889	18.91	1.8	3.8	1	18	27	0.46
European Journal of Clinical Microbiology and Infectious Diseases	45	1,286	28.58	3.0	7.3	1	22	35	0.55
Frontiers in Microbiology	37	509	13.76	4.5	8.5	2	14	21	1.40
Microbiology Spectrum	34	391	11.50	3.8	5.8	2	12	19	1.00
Plos One	33	1,225	37.12	2.6	5.4	2	22	33	1.47
Microbial Drug Resistance	26	497	19.12	1.9	6.2	2	15	22	0.50

ACI, Average Citations Per Item; JIF, Journal Impact Factor in 2024.

a, Scopus CiteScore 2024; b, Sources Zone were divided by Bradford's law; c, h-index, g-index, m-index were calculated based on modified Hindex function in Bibliometrix.

### 3.3 Author analysis

**Figure 4:**
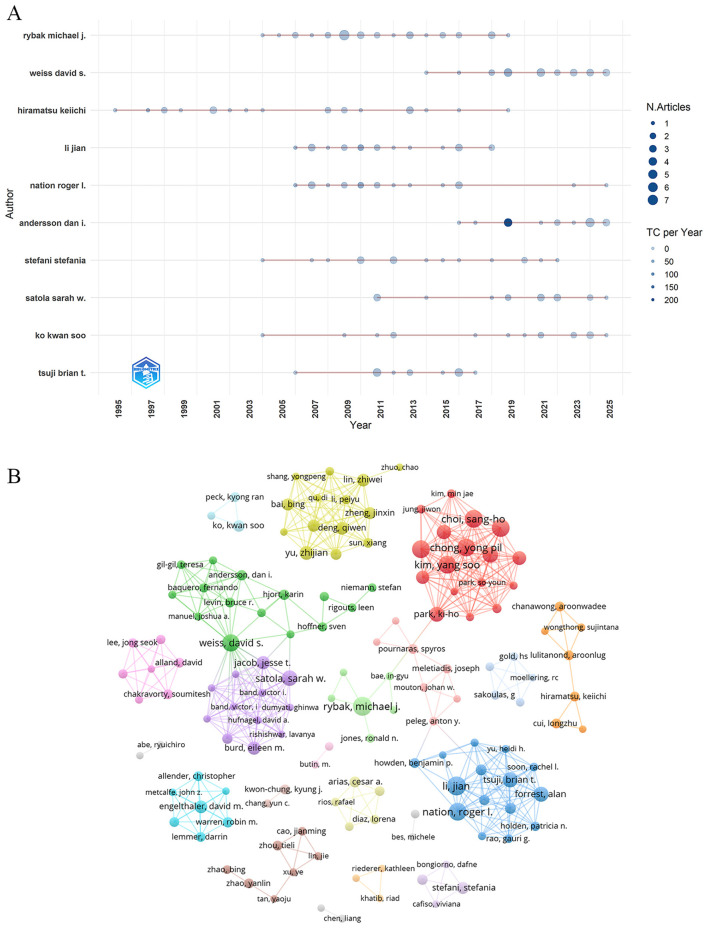
The author analysis of antibiotic heteroresistance in the merged collection. The top 10 active authors' production over time **(A)**; authors' co-authorship analysis **(B)** with at least 5 documents in the antibiotic heteroresistance merged collection.

The top 10 most active authors within the realm of antibiotic heteroresistance were detailed in Table 2. We determined that Michael J. Rybak (*n* = 32) from Wayne State University, David S. Weiss (*n* = 23) from Emory University, Keiichi Hiramatsu (*n* = 22) from Juntendo University, Jian Li (*n* = 19), and Roger L. Nation (*n* = 19) from Monash University were the top five most contributing authors in terms of the number of documents. The temporal changes in the number of publications and citations of the top 10 active authors in different years are shown in Figure 4. Most authors were active, with a minimum range of 6 years. Most authors, except Keiichi Hiramatsu, have been active since 2003, with a minimum active period of 10 years. In terms of total citations, the top five authors were Keiichi Hiramatsu, Jian Li, Roger L. Nation, Benjamin P. Howden, and Dan I. Andersson, each with more than 1,700 citations. In addition, the authors' co-citation analysis indicated that the most co-cited authors in both the WOSCC database and the Scopus database were Keiichi Hiramatsu. The author collaboration network (Figure 4) identified 21 author collaboration communities, with author collaborations presented as small-group clustering but lacking broad connectivity, where David S. Weiss had the greatest total link strength.

**Table 2:** Top 10 active authors on antibiotic heteroresistance research in merged collection.

Author name	Publications	Citations	Average citations per item	Affiliations
Rybak, Michael J.	32	1,439	44.97	Wayne State University
Weiss, David S.	23	846	36.78	Emory University
Hiramatsu, Keiichi	22	2,562	116.45	Juntendo University
Li, Jian	19	2,480	130.53	Monash University
Nation, Roger L.	19	2,428	127.79	Monash University
Andersson, Dan I.	18	1,718	95.44	Uppsala University
Stefani, Stefania	17	551	32.41	University of Catania
Satola, Sarah W.	16	549	34.31	Emory University
Ko, Kwan Soo	16	388	24.25	Sungkyunkwan University
Tsuji, Brian T.	14	700	50.00	University at Buffalo

### 3.4 Institution analysis

**Figure 5:**
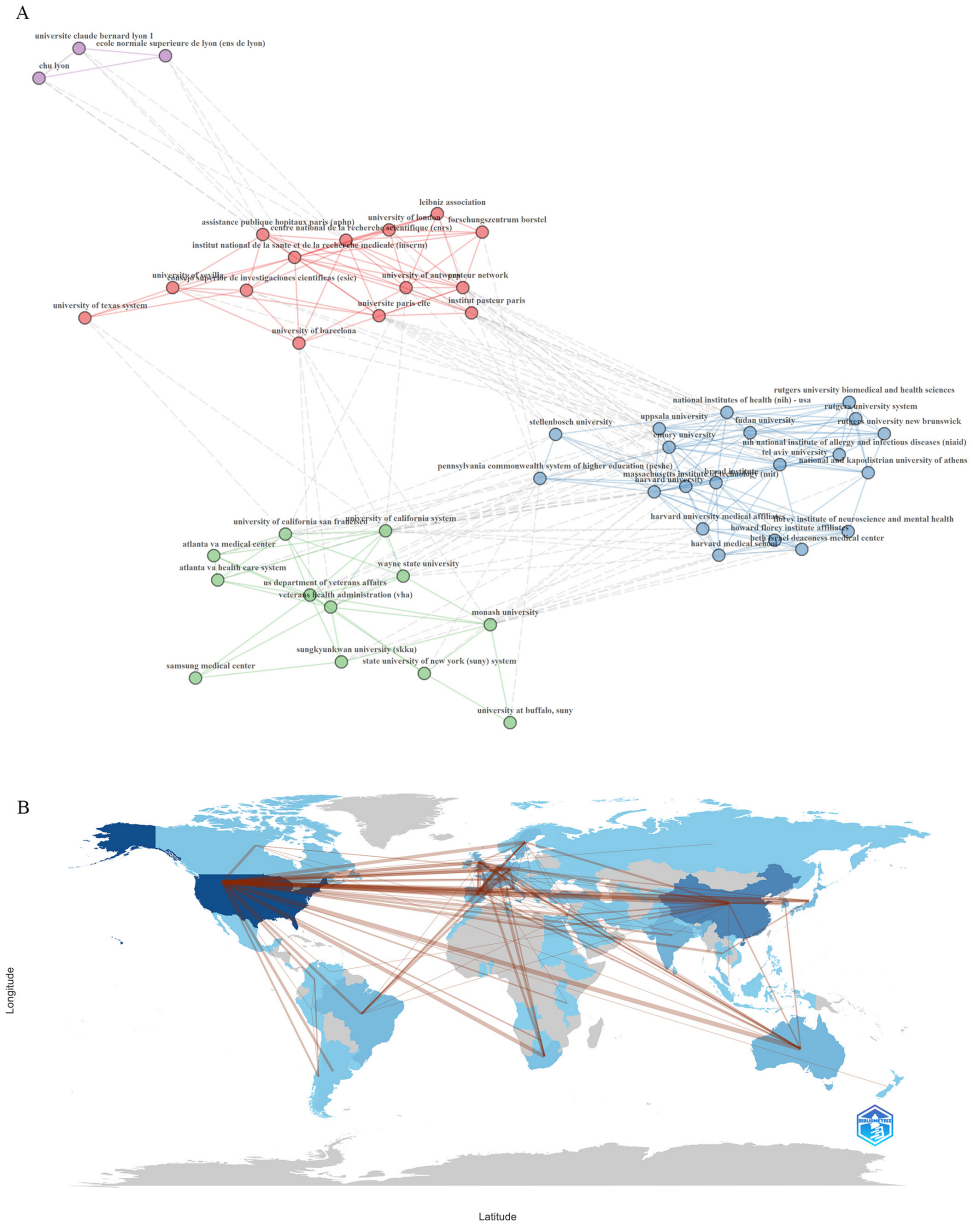
Institutions and Countries/regions' collaboration analysis of antibiotic heteroresistance in the merged collection. The institutions' collaboration network **(A)**; country collaboration map **(B)**.

Emory University was found to be the most productive institution (6.27% articles) followed by Wayne State University, Monash University, Harvard University, Sungkyunkwan University, Fudan University, University of California, University of Ulsan, Institut National De La Sante Et De La Recherche Medicale (INSERM), Universite Paris Cite in antibiotic heteroresistance research (Table 3); Monash University was the institution with the most citations. The co-authorship analysis of partnerships among organizations in merged collection manifested that four major communities comprising the top 50 institutions (Figure 5) were found and 13 super hub institutions, including Harvard University, Universite Paris Cite, Pasteur Network, University of California System, Emory University, Monash University, INSERM, Uppsala University, USA Veterans Health Administration (VHA), USA Department of Veterans Affairs, University of London, USA National Institutes of Health (NIH), Wayne State University, were identified by degree and between centrality.

**Table 3:** Top 10 active institutions on antibiotic heteroresistance research in the merged collection.

Institution	Publications	Citations	Average citations per item
Emory University	95	4,888	51.45
Wayne State University	59	2,467	41.81
Monash University	58	7,191	123.98
Harvard University	53	6,971	131.53
Sungkyunkwan University	52	1,122	21.58
Fudan University	50	993	19.86
University of California System	47	2,445	52.02
University of Ulsan	44	1,332	30.27
Institut National De La Sante Et De La Recherche Medicale (INSERM)	44	1,284	29.18
Universite Paris Cite	40	1,877	46.92

### 3.5 Countries analysis

Table 4 displayed the top 10 active countries in the merged collection of research on antibiotic heteroresistance, with the USA and China being the leading publishing countries, each contributing to over 200 publications. Among all contributing countries, the USA, Australia, China, Japan, and the United Kingdom ranked as the top five countries by total citation counts, and all with more than 2,000 citations. Australia had the highest average citations per article among the top 10 most active countries. The co-authorship analysis across all countries (Figure 5) revealed that the USA, the United Kingdom, France, Spain, and China ranked among the top five in total link numbers. The USA, the country with the largest number of publications, regularly collaborated with countries (more than 100 collaborations), including New Zealand, Japan, Australia, South Korea, Malaysia, Vietnam, China, and Thailand.

**Table 4:** Top 10 active Countries on antibiotic heteroresistance research in merged collection.

Country	Publications	Citations	Average citations per item
USA	332	14,919	44.94
China	216	4,257	19.71
India	70	771	11.01
Korea	61	1,430	23.44
France	57	1,634	28.67
Australia	51	4,808	94.27
United Kingdom	51	2,048	40.16
Spain	51	1,673	32.80
Brazil	50	864	17.28
Italy	47	1,362	28.98

### 3.6 Documents analysis

The top 10 cited articles and reviews in the mixed collection are detailed in Supplementary Tables S4, S5, respectively. The top 10 cited articles focused on the increasing glycopeptide resistance patterns in *S. aureus* (Tenover et al., 2001), the dissemination of heterogeneous vancomycin-intermediate *S. aureus* (hVISA) (Hiramatsu et al., 1997), the detection of hVISA using a modified PAP method (Wootton et al., 2001), the clinical features associated with hVISA bacteremia (Charles et al., 2004), the driving effect of the accessory gene regulator (agr) locus on vancomycin resistance (Sakoulas et al., 2002) the role of beta-lactamase in staphylococcal resistance (McDougal and Thornsberry, 1986), colistin heteroresistance in *A. baumannii* (Li et al., 2006) as well as the underlying mechanisms mediated by the complete loss of lipopolysaccharide production (Moffatt et al., 2010), integrating informatics tools and portable sequencing technology for rapid detection of resistance to antituberculous drugs (Phelan et al., 2019), and formation of disomy promoted *Cryptococcus neoformans to* overcome azole stress (Sionov et al., 2010). The top 10 cited reviews centered on core concepts of antimicrobial heteroresistance (El-Halfawy and Valvano, 2015; Andersson et al., 2019), antibiotic persistence (Balaban et al., 2019), vancomycin in *S. aureus* (Coia et al., 2006; Tenover and Moellering, 2007; Howden et al., 2010), colistin (Ahmed et al., 2020), colistin resistance of *A. baumannii* (Novovic and Jovcic, 2023), *H. pylori* infection and its antibiotic resistance (Tshibangu-Kabamba and Yamaoka, 2021), and *Pseudomonas aeruginosa* adaptation and evolution (Rossi et al., 2021).

### 3.7 Keyword analysis

**Figure 6:**
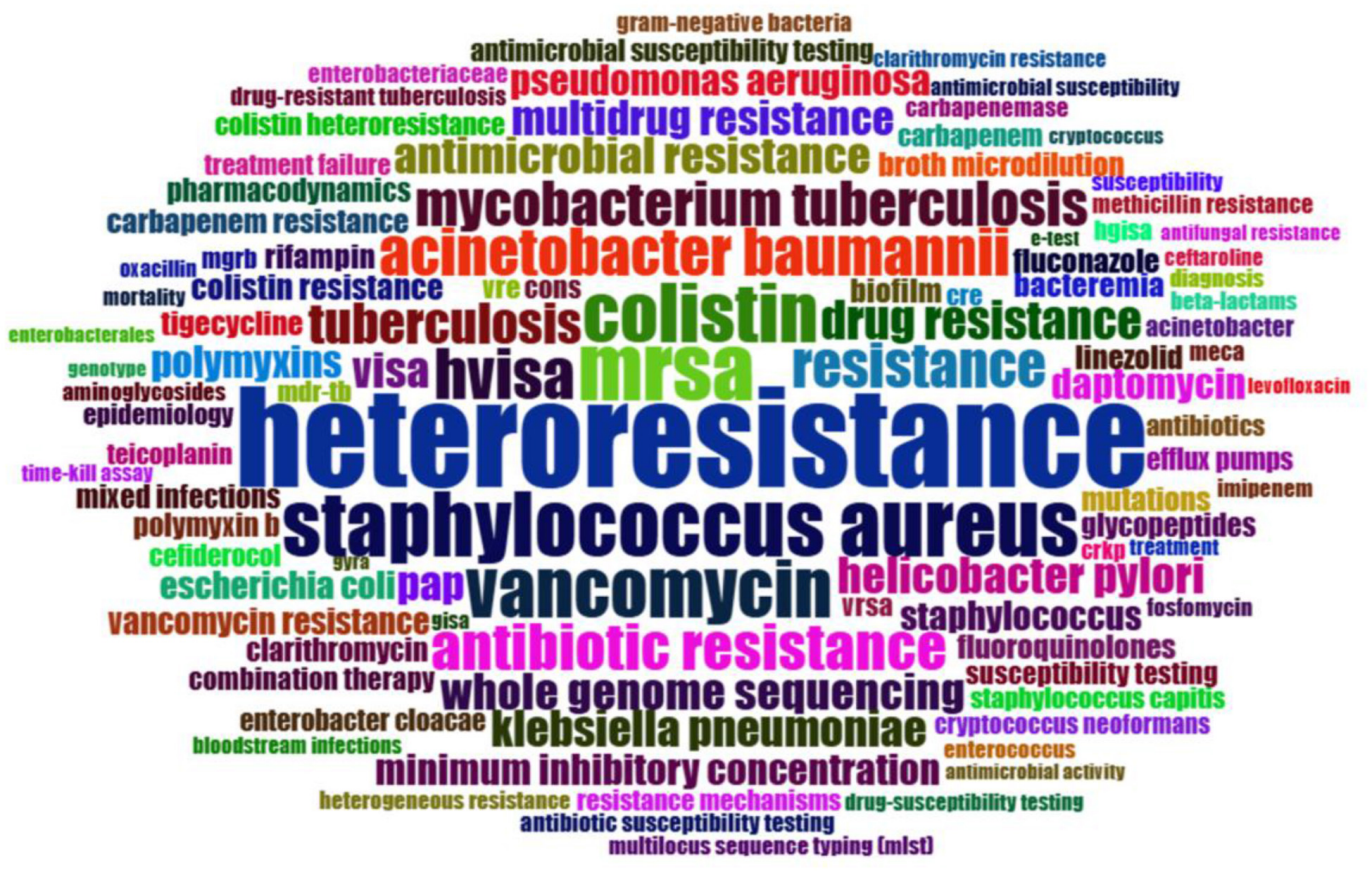
Word cloud of the top 100 author keywords of antibiotic hetero-resistance in the merged collection.

**Figure 7:**
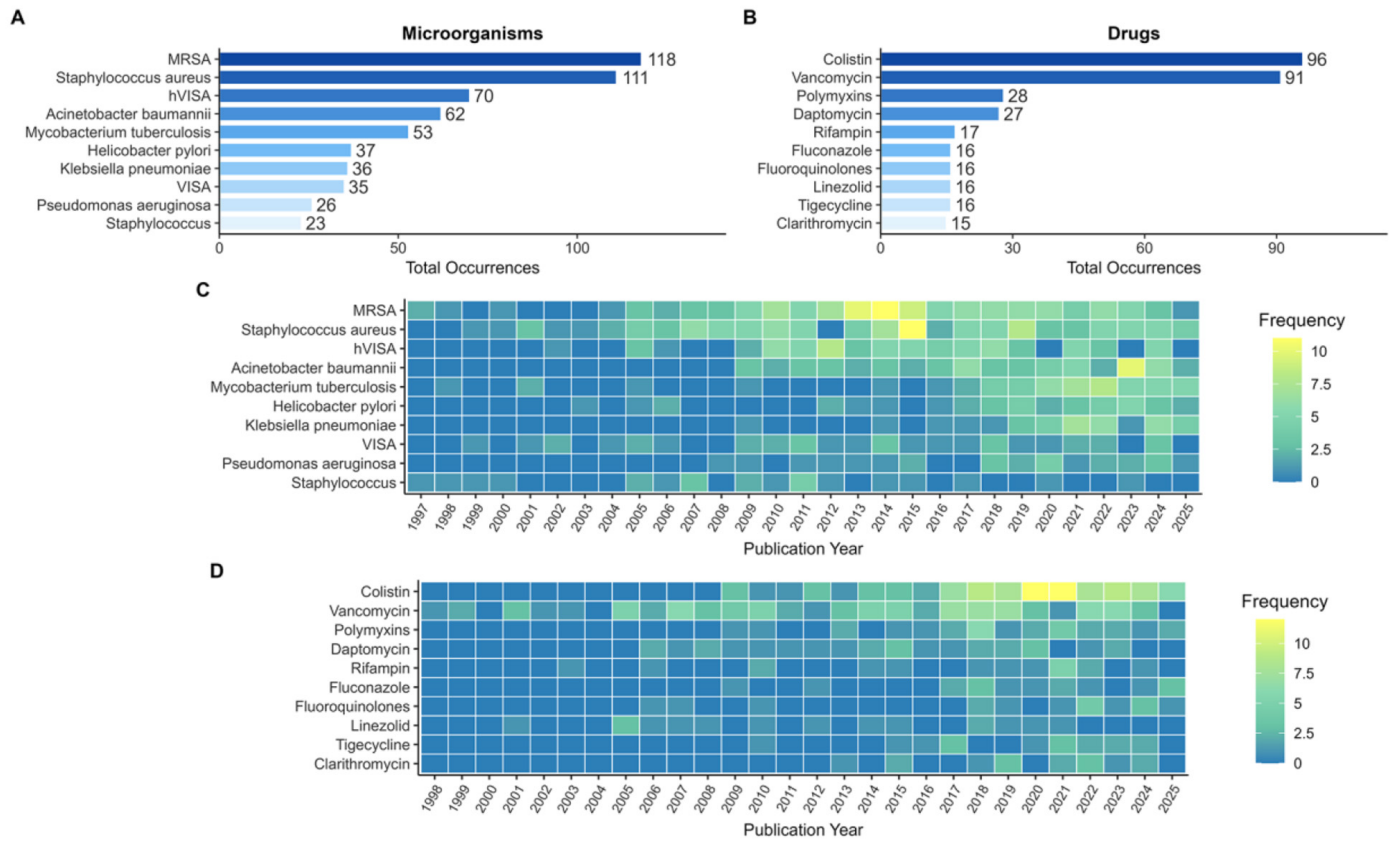
Top 10 author keywords related to microorganisms and antimicrobial drugs of antibiotic heteroresistance in the merged collection. The occurrence frequency of the top 10 author keywords of microorganisms **(A)** and antimicrobial drugs **(B)**. The heatmap of temporal changes in keyword frequency of the top 10 author keywords of microorganisms **(C)** and antimicrobial drugs **(D)**.

A total of 1,758 author keywords were extracted from 1,516 documents in the merged collection after merging of synonyms. The important author keywords on antibiotic heteroresistance research in the merged collection were shown in Table 5 (top 10 author keywords) and the word cloud Figure 6 (top 100 author keywords), respectively. In the study of antibiotic heteroresistance, the top 10 author keywords of microorganisms were methicillin-resistant *S. aureus* (MRSA), *Staphylococcus aureus*, hVISA, *Acinetobacter baumannii, Mycobacterium tuberculosis, Helicobacter pylori, Klebsiella pneumoniae*, vancomycin-intermediate *S. aureus* (VISA), *Pseudomonas aeruginosa, Staphylococcus*, and the top 10 author keywords of antimicrobial drugs were colistin, vancomycin, polymyxins, daptomycin, rifampin, fluconazole, fluoroquinolones, linezolid, tigecycline, and clarithromycin (Figure 7). Analysis of temporal changes in microorganisms-related keyword frequencies revealed that *S. aureus-*associated topics were the first to attract attention and remained prominent to date, while the frequency of common multidrug-resistant gram-negative bacteria (*A. baumannii, K. pneumoniae, P. aeruginosa*) gradually increased since 2009, and the attention to *M. tuberculosis* as well as *H. pylori* also increased significantly in the past 10 years (Figure 7). The temporal trends in the frequency of antibacterial drug keywords reflected that colistin and vancomycin have received the most attention for a long time (Figure 7). The reported heteroresistance in microorganism-antimicrobial combinations was summarized in Supplementary Table S6.

**Table 5:** Top 10 author keywords on antibiotic heteroresistance research in merged collection.

Rank	Author keywords	Occurrences	Betweenness	Closeness	Page rank
1	Heteroresistance	275	656.075	0.019	0.131
2	MRSA	118	61.868	0.013	0.057
3	Staphylococcus aureus	111	52.308	0.013	0.051
4	Colistin	96	23.326	0.013	0.052
5	Vancomycin	91	48.923	0.013	0.055
6	hVISA	70	5.987	0.011	0.035
7	Acinetobacter baumannii	62	30.399	0.013	0.036
8	Antibiotic resistance	61	14.554	0.013	0.029
9	Resistance	56	49.996	0.013	0.032
10	Mycobacterium tuberculosis	53	2.854	0.011	0.024

MRSA, Methicillin-resistant *Staphylococcus aureus*; hVISA, Heterogeneous vancomycin-intermediate *Staphylococcus aureus*.

The values of betweenness centrality, closeness centrality, and pagerank centrality were calculated by Bibliometrix based on all author keywords.

**Figure 8:**
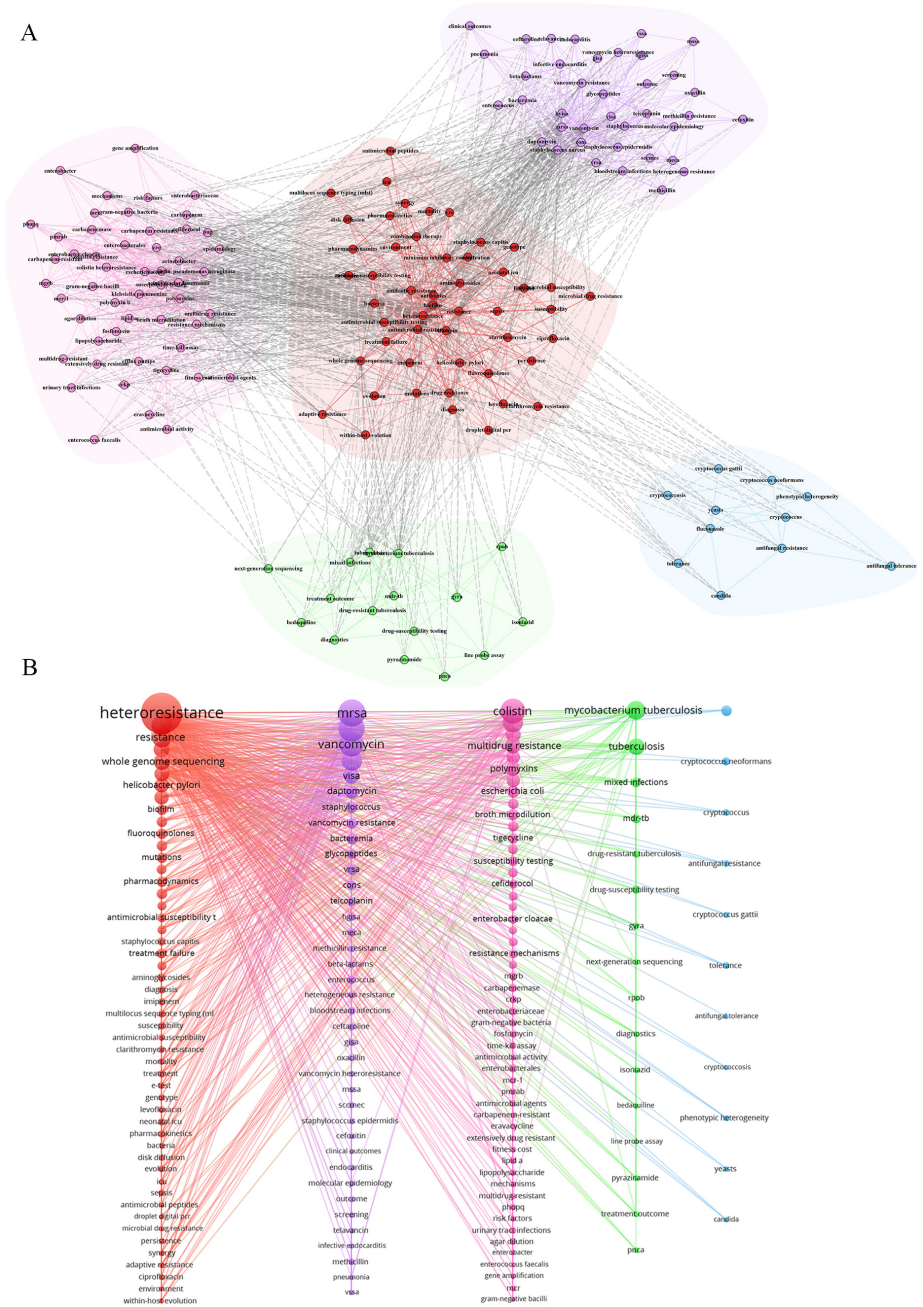
Keywords co-occurrence of antibiotic heteroresistance in the merged collection. The author keywords co-occurrence network was plotted by Bibliometrix **(A)** and VOSViewer **(B)**, which was divided into five clusters, and different colors represent different clusters.

When following the combinatorial default settings of VOSViewer and Bibliometrix (the minimum occurrence frequency of a keyword was set to five, normalization method chosen association method, and cluster type adapted walktrap algorithm) for keywords co-occurrence and clustering analysis, a total of 167 main keywords were identified and divided into five clusters (Figure 8). Cluster 1 (red) was associated with many common concepts related to antibiotic heteroresistance involving a total of 50 keywords, such as (i) resistance-associated basic terms (resistance, antibiotic resistance, antimicrobial resistance, drug resistance, adaptive resistance, microbial drug resistance, persistence, susceptibility, antimicrobial susceptibility); (ii) widely linked antimicrobial agents (antibiotics, imipenem, linezolid, aminoglycosides, quinolones, levofloxacin, ciprofloxacin, rifampin, clarithromycin, antimicrobial peptides); (iii) diagnosis and testing technology (diagnosis, pharmacodynamics, pharmacokinetics, antibiotic susceptibility testing, antimicrobial susceptibility testing, minimum inhibitory concentration, e-test, disk diffusion, whole genome sequencing, droplet digital PCR, genotype, multilocus sequence typing); (iv) disease treatment and outcomes (treatment, treatment failure, combination therapy, synergy, mortality, sepsis, ICU); (v) underlying mechanisms (mutations, biofilm, environment, evolution, within-host evolution). Cluster 2 (purple) had keywords mainly related to common multidrug-resistant Gram-positive bacteria (MDR-GPB), closely connected antibiotic agents, and other related content, like MRSA, hVISA, VISA, Vancomycin-resistant *S. aureus* (VRSA), heteroresistant glycopeptide intermediate *S. aureus* (hGISA), glycopeptides, vancomycin, daptomycin, teicoplanin, telavancin, beta-lactams, methicillin, oxacillin, cefoxitin, ceftaroline, including 38 keywords. Cluster 3 (pink) was related to common multidrug-resistant Gram-negative bacteria (MDR-GNB), therapeutic antimicrobial agents, and other associated terms, such as *A. baumannii, K. pneumoniae, P. aeruginosa, Escherichia coli, Enterobacter*

*cloacae*, carbapenem-resistant *Enterobacterales* (CRE), carbapenem-resistant *K. pneumoniae* (CRKP), colistin, polymyxins, polymyxin b, tigecycline, carbapenem, cefiderocol, fosfomycin, and eravacycline. Cluster 4 (green) dealt with *M. tuberculosis*, mainly involving therapeutic antimycobacterial drugs such as isoniazid, pyrazinamide, bedaquiline, diagnostic techniques, namely drug susceptibility testing, next-generation sequencing, line probe assay, as well as underlying mechanisms including mixed infections, rpoB, pncA, and gyrA. Cluster 5 (blue) is largely connected with antifungal heteroresistance, like *C. neoformans, Cryptococcus gattii, Candida*, yeasts, and fluconazole.

**Figure 9:**
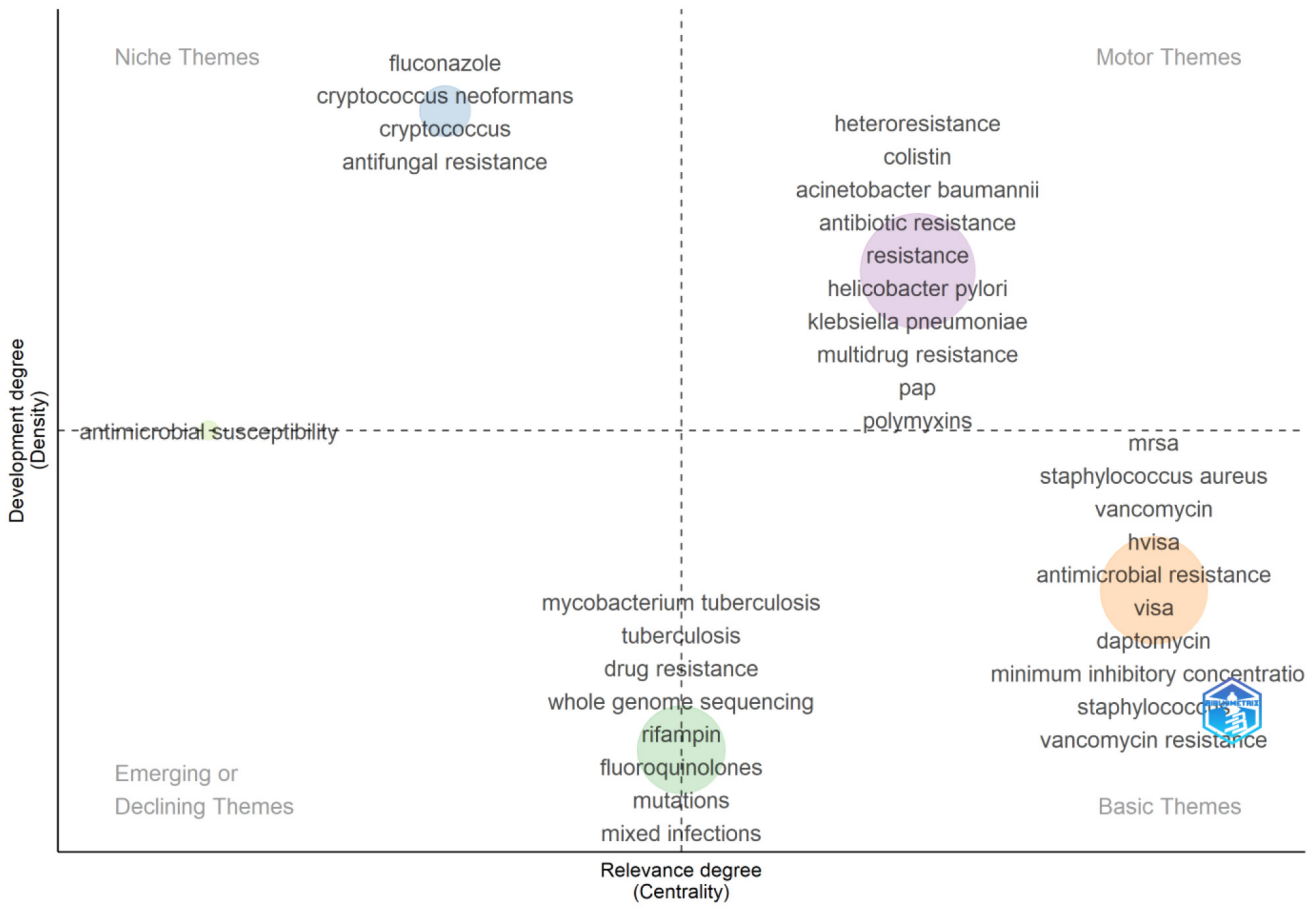
Thematic map of antibiotic heteroresistance in the merged collection.

The thematic map was constructed (Figure 9) based on a two-dimensional matrix, with centrality as the X-axis indicating the importance of a theme and density as the Y-axis indicating the development of a theme. The important and well-developed motor themes in the first quadrant involved antibiotic heteroresistance of multidrug-resistant Gram-negative bacteria like *A. baumannii, K. pneumoniae*, and *H. pylori*, with a particular focus on colistin and polymyxins. The niche themes in the second quadrant included fluconazole, *C. neoformans, Cryptococcus*, and antifungal resistance, indicating that these are highly developed, isolated themes. The circle includes *M. tuberculosis*, tuberculosis, drug resistance, whole genome sequencing, rifampin, fluoroquinolones, mutations, and mixed infections across the third and fourth quadrants, suggesting that they were both emerging themes and basic themes. The fourth quadrant items indicated the transversal and basic themes, mainly related to *S. aureus*, such as MRSA, hVISA, VISA, vancomycin, and daptomycin.

**Figure 10:**
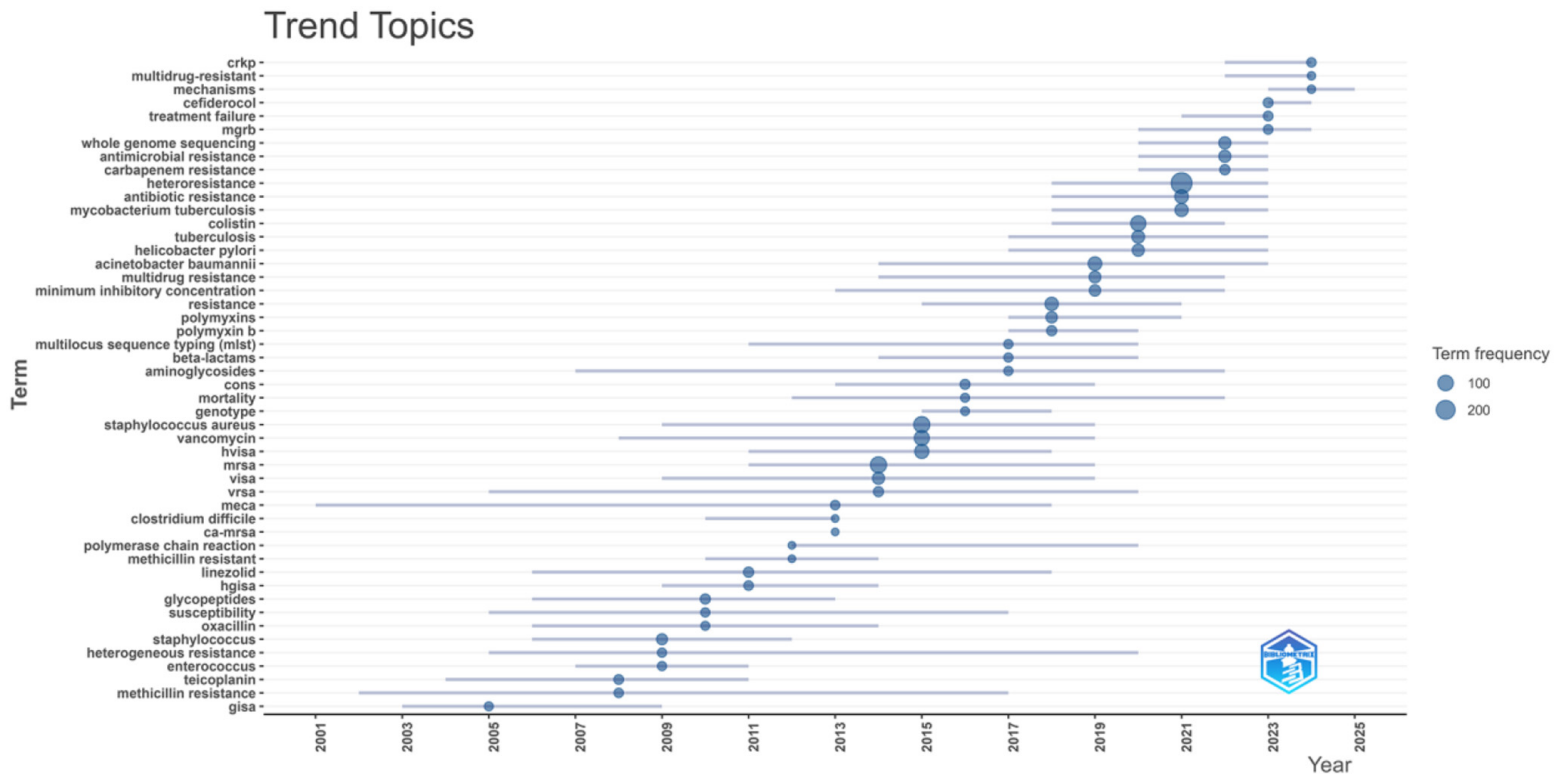
Keywords trend topics of antibiotic heteroresistance in the merged collection.

Moreover, a trend topics analysis based on authors' keywords was performed using Bibliometrix (Figure 10). The trend topics results demonstrated that bacteria have shifted from MDR-GPB represented by *S. aureus* (GISA, hGISA, MRSA, VRSA, VISA, hVISA) to *A. baumannii, H. pylori, M. tuberculosis*, CRKP, and the shift from antibiotics mainly used against gram-positive bacteria (oxacillin, methicillin, teicoplanin, glycopeptides, linezolid, vancomycin) to MDR-GNB related antimicrobial drugs(aminoglycosides, beta-lactams, polymyxins, polymyxin b, colistin, carbapenem, cefiderocol). Notably, hot keywords in the last 3 years included CRKP, multidrug-resistant, mechanisms, cefiderocol, treatment failure, mgrB, whole genome sequencing, antimicrobial resistance, and carbapenem resistance.

## 4 Discussion

### 4.1 General information

In this study, we presented the overall research trend of antibiotic heteroresistance over the past decades. The increasing number of publications and citations on antibiotic heteroresistance indicates a growing interest in antibiotic heteroresistance in different fields over time. The concept of heterogeneous antibiotic resistance was initially described in the 1940s for the Gram-negative bacterium *Haemophilus influenzae type b* (Alexander and Leidy, 1947) and later in the 1960s for Gram-positive staphylococci (Sutherland and Rolinson, 1964), yet the term “heteroresistance” was not actually reported until 1970 (Kayser et al., 1970). Our merged dataset showed that over the past few decades, a variety of bacteria and fungi have been reported to exhibit heteroresistance to different antimicrobial agents, as detailed in Supplementary Table S6. The significant time peak of the annual average number of citations revealed important research of common concern, including the clinical hVISA Mu3 strain reported in 1997, which was isolated from the sputum of a patient with pneumonia after surgery who had failed vancomycin therapy (Hiramatsu et al., 1997), colistin heteroresistance in multidrug-resistant *A. baumannii* was first reported in 2006 (Li et al., 2006) colistin hetero-resistance to *A. baumannii* could be due to the selection of lipid A biosynthesis mutants (Moffatt et al., 2010), definitions and guidelines for antibiotic persistence (Balaban et al., 2019), mechanisms and clinical relevance of bacterial heteroresistance (Andersson et al., 2019), *Helicobacter pylori* infection and its antibiotic resistance (Tshibangu-Kabamba and Yamaoka, 2021), and *P. aeruginosa* within-host adaptation and evolution (Rossi et al., 2021). The top 10 cited documents also highlighted these key concepts of antimicrobial heteroresistance, detection methods, clinical features, and underlying mechanisms.

Journal analysis showed that six core journals, including Antimicrobial Agents and Chemotherapy, Journal of Clinical Microbiology, Journal of Antimicrobial Chemotherapy, International Journal of Antimicrobial Agents, Diagnostic Microbiology and Infectious Disease, and European Journal of Clinical Microbiology & Infectious Diseases, belonged to the clinical microbiology or pharmacology pharmacy categories. In addition, open-access pan-microbiology journals have recently shown great interest in antimicrobial heteroresistance. This information of journal analysis will help scholars screen major journals with high-quality research related to antibiotic heteroresistance and select journals suitable for submission in this field.

The author analysis identified important research teams, which can help researchers quickly locate the core research findings on specific topics on antibiotic heteroresistance. Professor Keiichi Hiramatsu from Juntendo University, the most cited and co-cited author, first reported the clinical hVISA Mu3 strain (Hiramatsu et al., 1997) and conducted a series of studies on the potential mechanism of heterogeneous-to-homogeneous phenotypic transition of *S. aureus* resistance (Neoh et al., 2008; Katayama et al., 2009; Cui et al., 2010; Aiba et al., 2013; Matsuo et al., 2013). Professor Michael J. Rybak of Wayne State University in the USA led the publication count, focusing on topics of antibiotic heteroresistance in *S. aureus*, like hVISA (Rybak et al., 2008; Leonard et al., 2009a; Leonard and Rybak, 2009; Vidaillac et al., 2010; Steed et al., 2011, 2012; Werth et al., 2013; Smith et al., 2015; Claeys et al., 2016; Smith et al., 2016; Singh et al., 2018; Trinh et al., 2018; Xhemali et al., 2019), Hgisa (Leonard et al., 2009b; Lalani et al., 2008; Leuthner et al., 2006). His research team conducted extensive work using PK/PD models to evaluate the antibacterial activity of telavancin (Leonard et al., 2009b; Leuthner et al., 2006), ceftaroline (Vidaillac et al., 2010; Steed et al., 2011; Werth et al., 2013; Singh et al., 2018), oritavancin (Smith et al., 2016), dalbavancin (Xhemali et al., 2019), vancomycin (Leonard and Rybak, 2009; Steed et al., 2012; Trinh et al., 2018) alone or in combination with gentamicin or rifampin (Leonard et al., 2009b), tobramycin (Vidaillac et al., 2010), trimethoprim-sulfamethoxazole (Steed et al., 2012), b-lactams (Smith et al., 2016; Xhemali et al., 2019) for hVISA strains. From Emory University in the USA, with the leading number of publications, the research team of professor David S. Weiss has been studying heteroresistance to CRGNB, including *E. cloacae* (Lin C. K. et al., 2024; Band et al., 2019; Napier et al., 2014; Band et al., 2016), CRE (Lin C. K. et al., 2024; Band et al., 2019; Babiker et al., 2022; Band et al., 2021), CRKP (Wozniak et al., 2019; Band et al., 2018; Witt et al., 2022), carbapenem-resistant *P. aeruginosa* CRPA (Howard-Anderson et al., 2022), and antibiotics heteroresistance, such as colistin (Napier et al., 2014; Band et al., 2016; Babiker et al., 2022; Band et al., 2021; Wozniak et al., 2019; Band et al., 2018; Howard-Anderson et al., 2022), aminoglycoside (Anderson et al., 2018), β-lactam (Band and Weiss, 2021), β-lactam/β-lactamase inhibitors (Lin C. K. et al., 2024; Babiker et al., 2024) (ceftazidime/avibactam, imipenem/relebactam, meropenem/vaborbactam, and piperacillin/tazobactam). Professors Jian Li and Roger L. Nation from Monash University in Australia, top authors in publications, citations, and co-citations, were mainly committed to the research on colistin heteroresistance and polymyxin b heteroresistance in *A. baumannii* (Li et al., 2006; Moffatt et al., 2010; Soon et al., 2009; Owen et al., 2007; Tan et al., 2007; Lee et al., 2013; Dudhani et al., 2010; Yau et al., 2009; Rao et al., 2016a,b), *K. pneumoniae* (Poudyal et al., 2008; Deris et al., 2012), and *P. aeruginosa* (Ly et al., 2015; Bergen et al., 2011b,a), and have achieved notable accomplishments, particularly in the field of pharmacokinetics\pharmacodynamics (Tan et al., 2007; Dudhani et al., 2010; Yau et al., 2009; Poudyal et al., 2008), as well as in evaluating the synergistic antibacterial activity of polymyxins combined with doripenem (Rao et al., 2016a; Deris et al., 2012; Ly et al., 2015; Bergen et al., 2011b), imipenem (Bergen et al., 2011a), tigecycline (Rao et al., 2016b), or rifampin (Lee et al., 2013). They reported for the first time the existence of colistin heteroresistance in clinical strains of *A. baumannii* (Li et al., 2006), and also confirmed that loss of LPS due to the mutation of key lipid A biosynthesis genes can lead to colistin resistance and increased sensitivity to other clinically relevant antibiotics in a range of *A. baumannii* strains (Moffatt et al., 2010). Their modern polymyxin pharmacology data promoted practical guidelines for the optimal use of polymyxin (Nation et al., 2015; Tsuji et al., 2019), and significantly improved clinical practice worldwide.

The collaboration network of authors and institutions revealed that small group cooperation forms often focused on specific microorganisms rather than general themes. However, establishing a general consensus on heteroresistance may require extensive participation from research groups and institutions, indicating the need for broader collaboration in the future. In addition, the countries' collaboration network indicated inter-country cooperation seemed to be a core country-based cooperation model, which may be mainly driven by the main researchers.

### 4.2 Core research themes

The comprehensive analysis of keyword co-occurrence and clustering information allows us to accurately outline the research focus and scope of this field. In this study, we summarized five core clusters related to antibiotic heteroresistance, including common concepts related to antibiotic heteroresistance, common MDR-GPB, common MDR-GNB, *M. tuberculosis*, and antifungal heteroresistance. The thememap also identified the main themes of four major microoganisms-antimicrobials combinations.

#### 4.2.1 Heteroresistance terms cluster

For the first cluster with antibiotic heteroresistance, the definition and detection methods of heteroresistance would be the foundation of all associated studies. The broadest definition of antibiotic heteroresistance is the presence of a heterogeneous population of microorganisms with one subpopulation or several subpopulations that exhibit increased levels of antibiotic resistance compared with the main population (Andersson et al., 2019). Meanwhile, El-Halfawy OM et al. summarized some other heteroresistance definitions in detail (El-Halfawy and Valvano, 2015). Multiple definitions of heteroresistance have led to the phenomenon with many very different characteristics, making it impossible to retrospectively compare between different studies and to assess its true clinical significance (El-Halfawy and Valvano, 2015; Andersson et al., 2019). The levels of antibiotic resistance can be measured by various antimicrobial susceptibility testing, such as growth phenotype-based methods (for example, broth microdilution method, E-test, disk diffusion assays, agar dilution testing) and genetic-based molecular detection methods (for example, line probe assays, GeneXpert Xpert MTB/RIF Ultra, PCR, whole genome sequencing). For difficult-to-culture microorganisms, molecular detection methods of drug resistance levels played a major role, leading to differences in the definition of heteroresistance. Therefore, heteroresistance in *M. tuberculosis* was defined as the coexistence of antituberculosis drug-susceptible and -resistant bacteria in the same patient (Folkvardsen et al., 2013; Hofmann-Thiel et al., 2009), and based on molecular resistance detection, further defined as the coexistence of populations with differing nucleotides at a drug resistance locus within a sample of organisms (Eilertson et al., 2014). Based on the origin and clonality of resistant subpopulations, it can be divided into monoclonal heteroresistance and polyclonal heteroresistance (Andersson et al., 2019), which may be more conducive to reflecting the effects of different infection mechanisms and selecting more matching detection methods. From the perspective of microbiology laboratories testing and clinical treatment, Roch M et al. proposed a microbial definition of heteroresistance that refers to the detection of a resistant subpopulation from an overall susceptible isolate by standard minimal inhibitory concentration (MIC) assay, at a minimum frequency between 10^−8^ and 10^−6^, able to grow in the presence of an antibiotic concentration of at least two-fold the breakpoint (Roch et al., 2023). The microbial definition of heteroresistance highlighted the key technical points of population analysis profile (PAP), but also ignored the potential molecular resistance detection methods. We believed that the broad definition of heteroresistance may be intended to simply describe this phenomenon; however, a specific heteroresistance definition based on a species-antimicrobials combination involving phenotypic or molecular detection techniques may be more useful for detection and evaluation of clinical significance.

In terms of heteroresistance detection, E-test and disk diffusion assays were often used as preliminary screening methods due to the appearance of distinct colonies growing within the clear zone of inhibition, but false-positive and false-negative heteroresistance were found in both methods (Lo-Ten-Foe et al., 2007; Banerjee et al., 2024; van Hal et al., 2011). PAP test and its derivative methods were reliable methods for identifying heteroresistance (Wootton et al., 2001; van Hal et al., 2011), but the lack of standardization of operation, high cost, and high labor intensity hindered its implementation in clinical laboratories. Molecular detection methods can break through the limitations of culture conditions, but also rely on a comprehensive understanding of the genes and mutations related to drug resistance. Among difficult-to-culture microorganisms, such as *M. tuberculosis* and *H. pylori*, a variety of molecular methods were adapted to detect heteroresistance, such as line probe assays, droplet digital PCR, deepmelt assay, high-resolution melting, Sanger sequencing, targeted deep sequencing, and whole genome sequencing (Zhang et al., 2023; Whitfield et al., 2022; Liang et al., 2018; Kumar et al., 2014; Sun et al., 2018). In addition, a droplet microfluidics method with single-cell resolution was exploratory and successfully used to detect ampicillin heteroresistance in *E. coli* (Lyu et al., 2018), and a combination of WGS and machine learning can identify piperacillin/tazobactam heteroresistance in *E. coli* with perfect sensitivity and high specificity (Guliaev et al., 2025). The process and methods of heteroresistance detection would be the basis for determining the specific definition of heteroresistance in a given microorganism-antimicrobial combination, which is crucial for the realization of routine antibiotic heteroresistance testing in clinical laboratories, and is also an innovative direction that needs to be focused on in future research.

#### 4.2.2 Gram-positive bacteria

For the second cluster of common MDR-GPB, *Staphylococcus* and *Enterococcus* were representative microorganisms, involving commonly used antibiotics for gram-positive bacteria such as glycopeptides (vancomycin, teicoplanin, telavancin), daptomycin, beta-lactams (methicillin, oxacillin, cefoxitin, ceftaroline). The thematic map indicated that *S. aureus* and vancomycin constitute the basic theme of antibiotic heteroresistance. *S. aureus* can cause a wide variety of infections, and its adaptive power to antibiotics has resulted in the remarkable level of acquisition of resistance against multiple antibiotic classes, especially known for the global prevalence of MRSA (Deurenberg and Stobberingh, 2009; Lakhundi and Zhang, 2018). The rapid development of MRSA worldwide has led to the widespread clinical application of glycopeptides such as vancomycin, which is losing potency against MRSA, and clinical laboratories have gradually reported hVISA, VISA, VRSA, hGISA, and vancomycin heteroresistant *S. aureus* (hVRSA) associated with vancomycin treatment failure and increased mortality seen in patients with MRSA infection (Sakoulas and Moellering, 2008). The main sample source of hVISA was blood (60%), followed by lung (21%), skin and wound infections (14%), abscess (1%), and other (4%) (Rybak et al., 2008), suggesting the importance of assessing the association of hVISA with the clinical outcome of bloodstream infection and pneumonia. The previous retrospective cohort studies suggested that hVISA was independently associated with vancomycin treatment failure for *S. aureus* bloodstream infections (Casapao et al., 2013) and inpatient mortality was significantly higher in hVISA patients than in vancomycin-susceptible *S. aureus* pneumonia (Claeys et al., 2016). The prevalence of hVISA throughout the world ranges from 0% to 73.7% (van Hal and Paterson, 2011), and hVISA infection has increased continuously in the past decades (Keikha and Karbalaei, 2024). The clinical features and epidemiological characteristics of hVISA were often studied retrospectively, and retrospective bias may lead to differences in conclusions between studies. In the future, multicenter prospective clinical studies need to provide more reliable clinical significance assessments and more accurate prevalence results.

hVISA is the only type of heteroresistance with a clear consensus definition and a standardized detection assay recommended by EUCAST following the PAP protocol described by Wootton et al. (2001) Roch et al. (2023). The EUCAST defines hVISA as a *S. aureus* isolate susceptible to vancomycin (MIC ≤ 2 mg/L) but with minority populations (>10^−6^ cells) growing on vancomycin >2 mg/L by PAP confirmation (EDUCAST, 2017). hVISA strains appear to be the precursors of VISA strains and mutations affecting dozens of effector genes in diverse functionary categories that promote this hVISA to VISA phenotypic conversion have been described such as mutations in the two-component regulatory systems *vraSR* and *graRS* (Neoh et al., 2008; Cui et al., 2009), mutation in the *rpoB* gene (Matsuo et al., 2011), mutation in the *PP2C* phosphatase gene (Passalacqua et al., 2012). Moreover, hVISA/VISA strains exhibit many phenotypic changes, including thickened cell walls, reduced growth rate, PBP changes, reduction in autolytic activity, resistance to lysostaphin, and metabolic changes (Matsuo et al., 2013; Chen et al., 2013). Interestingly, some research promoted that the development of glycopeptide heteroresistance in *S. aureus* may be related to compromised agr function and reduced autolysis (Sakoulas et al., 2003; Schwaber et al., 2003), while other study suggested no association between agr dysfunction and the presence of hVISA (Butterfield et al., 2011). Besides hVISA, some reports have studied the occurrence of heteroresistance in *S. aureus* for oxacillin (Liu et al., 2021), ceftaroline (Saravolatz et al., 2014), teicoplanin (Rybak et al., 2005), daptomycin (Sakoulas et al., 2006; Ji et al., 2020), trimethoprim-sulfamethoxazole (Coelho et al., 2017), eravacycline (Zhang et al., 2018), omadacycline (Bai et al., 2019), mupirocin (Mutu et al., 2022), ciprofloxacin (Li et al., 2025), moxifloxacin (Iqbal et al., 2020), and gentamicin (Heidarian et al., 2024). Recent research revealed that the high prevalence of heteroresistance in *S. aureus* is mainly attributed to a multitude of point mutations in various chromosomal core genes, including those involved in membrane and peptidoglycan/ teichoic acid biosynthesis and transport, tRNA charging, menaquinone and chorismite biosynthesis and cyclic-di-AMP biosynthesis, which may be related to the low content of resistance genes in *S. aureus* and lack of repeat sequences that allow tandem gene amplification of these genes (Heidarian et al., 2024).

Moreover, vancomycin heteroresistance in coagulase-negative staphylococci has been gradually reported (Szemraj et al., 2023; Bathavatchalam et al., 2021; Chong et al., 2016; Nunes et al., 2006), and its certain characteristics may be similar to those of hVISA, but more studies are needed for further validation. *Enterococcus faecalis* heteroresistance to vancomycin (Cárdenas et al., 2014), cefotaxime (Mainardi et al., 1998), tigecycline (Bai et al., 2022), eravacycline (Wen et al., 2020), omadacycline (Lin et al., 2020) and *Enterococcus faecium* heteroresistance to vancomycin (Alam et al., 2001; Khan et al., 2008; Chacko et al., 2018; Zhou et al., 2020; Sun et al., 2024), linezolid (Chacko et al., 2018), daptomycin (Chacko et al., 2018), teicoplanin (Qu et al., 2009), eravacycline (Wen et al., 2022) were reported, and their underlying molecular mechanism of hetero resistance may be related to mutations of drug target sites and increased expression of efflux pumps under antibiotic stress (Bai et al., 2022; Chacko et al., 2018; Sun et al., 2024). *Streptococcus pneumoniae* heteroresistance to telithromycin (Rantala et al., 2006), erythromycin (Lohsen and Stephens, 2023), penicillin (Morand and Mühlemann, 2007; Engel et al., 2014), cephalexin (Sorg and Veening, 2015), fosfomycin (Engel et al., 2013) had also been reported, and similarly, some mutations and drug efflux pumps may play a key role in conferring heteroresistance. The mechanism of heteroresistance in specific bacteria and drug combinations still needs to be clarified by combining multiple techniques such as genomics, proteomics and phenotypic testing.

#### 4.2.3 Gram-negative bacteria

The third cluster primarily contains keywords connected with common MDR-GNB and optional antibiotics for its treatment, such as CRE, *A. baumannii, K. pneumoniae, P. aeruginosa, E. coli*, colistin, polymyxins, tigecycline, eravacycline, carbapenem, cefiderocol, and fosfomycin. Heteroresistance to colistin and polymyxins in *A. baumannii* and *K. pneumoniae* was the motor theme of antibiotic heteroresistance research, with a significant increase in keyword frequency in the past decade. Trend topics analysis of keywords revealed that carbapenem resistance was the most frequent trend topic in recent years, reflecting that carbapenem-resistant Gram-negative bacteria (CR-GNB) in this cluster represent a key focus of recent antibiotic heteroresistance research. Notably, among Gram-negative bacteria, the increase in annual mortality due to CR-GNB far exceeded that of any other class of resistant bacteria over the same period (Tian et al., 2024). The heteroresistance of the mainstream and novel treatment options for CR-GNB, such as polymyxins, tigecycline, eravacycline, cefiderocol, and ceftazidime/ avibactam, may be one of the reasons for its clinical treatment failure, making it a current research hotspot. The underlying molecular mechanisms of heteroresistance are limited in research data and have not been fully elucidated. The existing studies mainly involved drug target-related resistant mutations, drug efflux pumps, biofilm formation, gene amplifications, and other perspectives, which still need to be comprehensively studied.

The pooled prevalence of colistin heteroresistance in *A. baumannii* was 33%, with considerable heterogeneity (Karakonstantis and Saridakis, 2020). Polymyxin B heteroresistance was found in 36% of the *A. baumannii* clinical isolates (Genteluci et al., 2020). More importantly, about 80% colistin heteroresistance isolates can evolve into a resistant phenotype following colistin exposure and withdrawal, which could lead to higher rates of treatment failure and contribute to the reservoir of colistin-resistant pathogens in health care settings (Kon et al., 2023). The prevalence of tigecycline heteroresistance in *A. baumannii* was about 56.2%~59.5% in South Korea (Jo and Ko, 2021). 17.36% eravacycline heteroresistant isolates were detected among carbapenem-resistant *A. baumannii* (CRAB) isolates with eravacycline MIC values ≤ 4 mg/L in China (Li et al., 2024). The prevalence of cefiderocol heteroresistance in *CRAB* isolates ranged 47.4%~59% (Longshaw et al., 2023; Shields et al., 2024). Heteroresistance to penicillins, cephalosporins, carbapenems, and aminoglycosides in *A. baumannii* was also described (Anderson et al., 2018; Hung et al., 2012; Ikonomidis et al., 2009; Cuenca et al., 2012; Harmer et al., 2022). However, the correlation between heteroresistance in *A. baumannii* and clinical outcomes remains controversial (Kon et al., 2023; Longshaw et al., 2023; Shields et al., 2024; Zulfiqar et al., 2025; Jo et al., 2023), requiring extensive evaluation through multicenter, high-quality, prospective clinical studies in the future. Nonetheless, a genomics study unveiled that heteroresistant *A. baumannii* clones may spread from country to country through medical institutions (André et al., 2024), which called for an early assessment of the potential spread risk of heteroresistance. Moreover, almost all reported colistin resistance mechanisms in *A. baumannii* are chromosomally mediated by mutations in the pmrAB two-component regulatory systems, mutations in lpxA, lpxC, and lpxD, or efflux pump mechanisms (Chen et al., 2020). Not surprisingly, chromosomal mutations (predominantly in PmrAB two-component system and lpx genes) resulting in lipopolysaccharide modification or loss, or overexpression of efflux pumps were found in resistant subpopulations of colistin heteroresistant *A. baumannii* isolates (Karakonstantis and Saridakis, 2020; Chen et al., 2020; Cafiso et al., 2021; Rodriguez et al., 2019; Machado et al., 2018; Charretier et al., 2018). In addition, insertion sequence (IS) elements seemed to be involved in heteroresistance of *A. baumannii* to antibiotics such as tigecycline (Jo and Ko, 2021; Jo et al., 2024), eravacycline (Li et al., 2024), tobramycin (Harmer et al., 2022) and imipenem (Lee et al., 2011) through upregulation of efflux pumps or β-lactamase. Gene amplifications (for example, a gene that encodes an efflux pump, an antibiotic-modifying enzyme, an antibiotic-degrading enzyme, or an antibiotic target-modifying enzyme) are one well-documented cause of heteroresistance (Nicoloff et al., 2019), such as colistin (Machado et al., 2018) and aminoglycoside (Anderson et al., 2018) heteroresistance in *A. baumannii*.

Colistin heteroresistance was detected in 10.1% of CRE isolates in the USA, and more than 90% of these heteroresistant isolates were classified as colistin susceptible by standard clinical diagnostic testing (Band et al., 2021). Among all the *Enterobacterales*, research on heteroresistance of *K. pneumoniae* has attracted the most interest, with the largest number of publications. The pooled prevalence of heteroresistant *K. pneumoniae* was 31.5% with substantial heterogeneity (Khoshnood et al., 2025). Heteroresistance to ampicillin, cefazolin, ceftazidime, cefepime, aztreonam, imipenem, meropenem, amoxicillin/clavulanate, piperacillin/tazobactam, cefoperazone/sulbactam, ceftazidime /avibactam, amikacin, ciprofloxacin, fosfomycin, tigecycline, and polymyxin B in *K. pneumoniae* was investigated simultaneously in China, in which the prevalence of heteroresistance varied across antibiotics, ranging from 1.5% to 85.1% (Zhang et al., 2025). Some animal models revealed that heteroresistance in *K. pneumoniae* can lead to treatment failure (Band et al., 2018; Xiong et al., 2021; Zhang et al., 2021), emphasizing the importance of identification of heterresistant isolates in clinical settings. Meanwhile, appropriate antibiotic combinations should be considered for eradicating heteroresistant bacterial infections (Lin J. Y. et al., 2024; Stojowska-Swedrzynska et al., 2022). More clinical studies related to CRKP heteroresistance would be needed to comprehensively understand its impact on clinical outcomes and provide guidance on treatment decision-making. In most heteroresistant isolates, mutations and insertions were detected, particularly in genes associated with the drug target sites, efflux pumps, production of lipopolysaccharide, and membrane permeability (Lin J. Y. et al., 2024). To fully better understand the associated mechanisms of heteroresistance *K. pneumoniae*, two specific reviews are recommended (Lin J. Y. et al., 2024; Stojowska-Swedrzynska et al., 2022). In addition, small colony variants, sub-populations within biofilms, could trigger heteroresistance to colistin in *K. pneumoniae* (Silva et al., 2016). Furthermore, heteroresistance to multiple antibiotics in other *Enterobacterales*, including *E. coli, Salmonella enterica*, and *E. cloacae complex*, was also identified, as detailed in the Supplementary material. The mechanisms of these heteroresistant bacteria were complex, involving coordinated regulation of multiple genes and gene amplifications (Nicoloff et al., 2019; Wei et al., 2025; Nicoloff et al., 2024; Milleville et al., 2024; Pereira et al., 2021; Kuang et al., 2020). Further research on the molecular mechanism of heteroresistance in CRE still needs to be carried out, because existing studies cannot support the molecular detection of heteroresistance in these microorganisms.

Clinical studies and epidemiological data on heteroresistance in *P. aeruginosa* were relatively scarce, with some previous data observing a high incidence of meropenem (72.5%) (He et al., 2018), imipenem (54.3%) (He et al., 2018), cefpenem (57.3%) (Jia et al., 2020), and colistin (26%) (Howard-Anderson et al., 2022) heteroresistance. Although some reports have pointed to the possible emergence of heteroresistant *P. aeruginosa* isolates during treatment with therapeutic drugs (Jia et al., 2020; Teran et al., 2024), the association of clinical outcomes with heteroresistance in *P. aeruginosa* has not been extensively studied. Previous studies suggested that down-regulated expression of the *OprD* porin, overexpression of efflux systems, and intrinsic *AmpC* β-lactamase or metallo-β-lactamases contributed to imipenem heteroresistance in *P. aeruginosa* (He et al., 2018; Xu et al., 2020; Mei et al., 2015; Ikonomidis et al., 2008). *AmpC* hyperproduction contributed to the development of cefepime heteroresistance in *P. aeruginosa* (Jia et al., 2020). Mutations in specific genetic loci for lipid A synthesis and regulation of modifications to lipid A, such as two-component regulatory systems (*PhoPQ* and *PmrAB*) were associated with colistin heteroresistance in *P. aeruginosa* (Lin et al., 2019), and even localized *pmrB* hypermutation could drive the evolution of colistin heteroresistance (Kapel et al., 2022). The upregulation of key genes involved in DNA replication and repair, and homologous recombination could cause unstable heteroresistance to levofloxacin in *P. aeruginosa* (Li W. R. et al., 2022). Cefiderocol heteroresistance was associated with mutations in the chromosomal cephalosporinase along with mutations in the *PirA* and *PiuA/D TonB*-dependent receptor pathways (Teran et al., 2024; Egge et al., 2024). Down-regulation of quorum-sensing-associated genes *lasI* and *rhlI* enhanced heteroresistance of *P. aeruginosa* to meropenem, amikacin, ciprofloxacin, and ceftazidime (Lu et al., 2022).

#### 4.2.4 Mycobacterium tuberculosis

The fourth cluster is chiefly composed of keywords concerning *M. tuberculosis*, antituberculosis drugs, and drug susceptibility testing techniques, which involved both basic and emerging themes in the thematic map. As mentioned earlier, the concept of heteroresistance in *M. tuberculosis* requires careful clarification. While true classic heteroresistance (i.e., the presence of a resistant subpopulation within a single *M. tuberculosis* clone) can occur, the term is often broadly used to describe the coexistence of drug-susceptible and drug-resistant populations within the same individual. This phenomenon may include both clonal heterogeneity (consistent with the classic definition of heteroresistance) and polyclonal coexistence of distinct strains (e.g., mixed infections or divergent lineages arising from within-host evolution) in a single patient sample, manifesting as populations with different nucleotides at drug resistance loci (Hofmann-Thiel et al., 2009; Figueredo et al., 2020; Rigouts et al., 2019; Mascellino et al., 2017). Heteroresistance in *M. tuberculosis* infection is common, and major antituberculosis drug heteroresistance has been reported in *M. tuberculosis*, but the prevalence varies between different studies and regions. The pooled prevalence of heteroresistance to isoniazid, rifampin, fluoroquinolones, and ethambutol were 5%, 7%, 10%, and 1%, respectively (Ye et al., 2021). The prevalence of pyrazinamide heteroresistance was lower than that identified for other first-line antituberculosis drugs (Whitfield et al., 2022). Amikacin heteroresistance was identified in 10.9% phenotypic amikacin-resistant *M. tuberculosis* isolates (Zhang et al., 2013). Besides, 21.05%~29% bedaquiline heteroresistance in *M. tuberculosis* was reported in Asia (Madadi-Goli et al., 2024; Rashid et al., 2025). Heteroresistance has been related to poor treatment outcomes of *M. tuberculosis* infection, such as persistent infection and treatment failure in multi/extensively drug-resistant (MDR/XDR) tuberculosis (Sonnenkalb et al., 2021). However, some studies suggested that heteroresistance is not associated with treatment outcomes of tuberculosis (Chen et al., 2021; Shin et al., 2018), but these studies had small sample sizes and lacked dynamic monitoring. The focus of future clinical research may take into account the dynamic monitoring of heteroresistance during tuberculosis treatment and the impact of therapeutic drug changes on clinical outcomes.

#### 4.2.5 Antifungal heteroresistance

The fifth cluster is mainly made up of keywords relevant to fungi and antifungal drugs, like *C. neoformans, C. gattii*, azoles, and fluconazole, which are located in a niche theme within the thematic map. From National Institutes of Health in the USA, Professor Kyung J. Kwon-Chung, a pioneer representative of antifungal heteroresistance, has long been committed to the azoles heteroresistance to *C. neoformans* and *C. gattii* species complexes, and the results of her team's study suggested that azoles heteroresistance in *C. neoformans* and *C. gattii* is an intrinsic adaptive resistance to azoles and the heteroresistant phenotype is associated with disomic chromosomes (D'Souza et al., 2011; Sionov et al., 2013), involving azoles target gene *ERG11* (Sionov et al., 2010), efflux pump-coding genes (Chang et al., 2018), critical genes required for maintenance of endoplasmic reticulum integrity (Ngamskulrungroj et al., 2012a,b). In addition, a previous study has demonstrated that *Candida spp*. could generate antifungal heteroresistance to fluconazole and amphotericin B by selection or induction *in vitro* (Claudino et al., 2009). Heteroresistance to fluconazole in *Candida glabrata* has also been reported, which may be associated with increased expression of genes that encode energy-dependent drug efflux transporters and determined by a combination of genetic and epigenetic determinants (Ben-Ami et al., 2016). *In vitro* evolution of *C. glabrata* populations on a gradient of caspofungin concentrations confirmed that resistance fitness cost in heterogeneous resistant subgroups did not result in reduced virulence, which may prompt the clinical importance of antifungal heteroresistance (Duxbury et al., 2020). Unfortunately, a recent study demonstrated that micafungin heteroresistance can cause antifungal prophylaxis failure in allogeneic hematopoietic cell transplantation patients and facilitate breakthrough *Candida parapsilosis* bloodstream infection (Zhai et al., 2024). The voriconazole heteroresistance in *Trichosporon asahii* clinical isolates was reported in China (Liu et al., 2025). To speed up the detection of antifungal heteroresistance in yeasts, a framework of population analysis profiling based on growth on solid medium, single-cell assays based on growth in liquid culture, and disk diffusion assays for the identification and measurement of heteroresistance in azole-sensitive *Candida* isolates has been developed (Gautier et al., 2024).

### 4.3 Strengths and limitations

In this study, we used Bibliometrix and VOSviewer to conduct a comprehensive bibliometric analysis of antibiotic heteroresistance studies from WOSCC, Scopus, and the PubMed database. The combined use of Bibliometrix and VOSviewer could give full play to the analytical performance advantages of each software and avoid deviations caused by analytical tools. Multi-source database analysis makes bibliometric results more objective and accurate, which allows quantitative literature analysis to provide a more comprehensive understanding of changing research priorities than narrative reviews. Inevitably, there are still some limitations in this study. Firstly, the articles included in this study were limited to those published in English, which may not rule out the impact of language publication bias. Secondly, the difference in update frequency between different databases may affect the number of retrieved literature.

## 5 Conclusion

As far as we know, our research is the first bibliometric study to conduct a scientific and systematic analysis of research trends and hotspots in antibiotic heteroresistance over the past few decades. This study can help researchers, microbiologists, and clinicians understand the profiles, trends, and key topics of antibiotic heteroresistance research and promote the integration of knowledge structures and the selection of emerging research directions.

## Data Availability

The raw data supporting the conclusions of this article will be made available by the authors, without undue reservation.
