# Supplementary material for: Global trends and research profile of antibiotic heteroresistance: a bibliometric and visual analysis
Source: Front Microbiol. 2026 Jul 17;17:1723669. doi: 10.3389/fmicb.2026.1723669 (PMC13424242; doi:10.3389/fmicb.2026.1723669)
Supplement: Supplementary file 1 [file Data_Sheet_1.pdf]

Supplementary Material

Supplementary Tables

Supplemental Table S1. Summary of Data Retrieval and Limited Field from the Web of Science Core Collection Database

**Category**	**Specific Standard requirement**
Database	Web of Science core collection
Time Span	From 1900-01-01 to 2025-06-30
Search Formula	TS=(Heteroresistan* OR heteroresistan* OR Hetero-resistan* OR hetero-resistan* OR "Heterogeneous resistan*" OR "heterogeneous resistan*" OR "Heterogeneous* resistan*" OR "heterogeneous* resistan*" OR "Heterogeneous* intermediate" OR "heterogeneous* intermediate" OR "heterogeneous vancomycin-intermediate" OR "heterogeneous glycopeptide-intermediate")
Language	English
Document Types	"Article" OR "Review Article"

Supplemental Table S2. Summary of Data Retrieval and Limited Field from the Scopus Database

**Category**	**Specific Standard requirement**
Database	Scopus
Time Span	PUBYEAR < 2026
Search Formula	TITLE-ABS-KEY (Heteroresistan* OR heteroresistan* OR Hetero-resistan* OR hetero-resistan* OR "Heterogeneous resistan*" OR "heterogeneous resistan*" OR "Heterogeneous* resistan*" OR "heterogeneous* resistan*" OR "Heterogeneous*-resistan*" OR "heterogeneous*-resistan*" OR "Heterogeneous* intermediate" OR "heterogeneous* intermediate" OR "Heterogeneous*-intermediate" OR "heterogeneous*-intermediate" OR "heterogeneous vancomycin-intermediate" OR "heterogeneous glycopeptide-intermediate")
Language	English
Document Types	"Article" OR "Review"

Supplemental Table S3. Summary of Data Retrieval and Limited Field from the PubMed Database

**Category**	**Specific Standard requirement**
Database	PubMed
Time Span	From 1900-01-01 to 2025-06-30
Search Formula	("heteroresistan*"[Title/Abstract] OR "heteroresistan*"[Title/Abstract] OR "hetero resistan*"[Title/Abstract] OR "hetero resistan*"[Title/Abstract] OR "heterogeneous resistan*"[Title/Abstract] OR "heterogeneous resistan*"[Title/Abstract] OR "heterogeneous* resistan*"[Title/Abstract] OR "heterogeneous* resistan*"[Title/Abstract] OR "heterogeneous* intermediate"[Title/Abstract] OR "heterogeneous* intermediate"[Title/Abstract] OR "heterogeneous vancomycin-intermediate"[Title/Abstract] OR "heterogeneous glycopeptide-intermediate"[Title/Abstract])
Language	English
Publication Type	"journal article" OR "review"

Supplemental Table S4. Top 10 cited articles on antibiotic heteroresistance research in merged collection.

Title	Citations	First Author	Journal Abbreviation	Year
Dissemination in Japanese hospitals of strains of Staphylococcus aureus heterogeneously resistant to vancomycin	989	Hiramatsu, K	Lancet	1997
Colistin Resistance in Acinetobacter baumannii Is Mediated by Complete Loss of Lipopolysaccharide Production	627	Moffatt, J	Antimicrob. Agents Chemother.	2010
Heteroresistance to colistin in multidrug-resistant Acinetobacter baumannii	458	Li, Jian	Antimicrob. Agents Chemother.	2006
Clinical Features Associated with Bacteremia Due to Heterogeneous Vancomycin-Intermediate Staphylococcus aureus	347	Charles, P	Clin. Infect. Dis.	2004
A modified population analysis profile (PAP) method to detect hetero-resistance to vancomycin in Staphylococcus aureus in a UK hospital	334	Wootton, M	J. Antimicrob. Chemother.	2001
Accessory gene regulator (agr) locus in geographically diverse Staphylococcus aureus isolates with reduced susceptibility to vancomycin	308	Sakoulas, G	Antimicrob. Agents Chemother.	2002
Increasing resistance to vancomycin and other glycopeptides in Staphylococcus aureus	293	Tenover, FC	Emerg. Infect. Dis	2001
Phosphoethanolamine Modification of Lipid A in Colistin-Resistant Variants of Acinetobacter baumannii Mediated by the pmrAB Two-Component Regulatory System	292	Beceiro, A	Antimicrob. Agents Chemother.	2011
Integrating informatics tools and portable sequencing technology for rapid detection of resistance to anti-tuberculous drugs	278	Phelan, J.	Genome Med.	2019
The role of beta-lactamase in staphylococcal resistance to penicillinase-resistant penicillins and cephalosporins	275	Mcdougal, LK	J. Clin. Microbiol.	1986
Cryptococcus neoformans Overcomes Stress of Azole Drugs by Formation of Disomy in Specific Multiple Chromosomes	275	Sionov, E	Plos Pathog.	2010

Supplemental Table S5. Top 10 cited reviews on antibiotic heteroresistance research in merged collection.

Title	Citations	First Author	Journal Abbreviation	Year
Definitions and guidelines for research on antibiotic persistence	829	Balaban, Nathalie Q.	Nat. Rev. Microbiol.	2019
Reduced Vancomycin Susceptibility in Staphylococcus aureus, Including Vancomycin-Intermediate and Heterogeneous Vancomycin-Intermediate Strains: Resistance Mechanisms, Laboratory Detection, and Clinical Implications	724	Howden, Benjamin P.	Clin. Microbiol. Rev.	2010
Colistin and its role in the Era of antibiotic resistance: an extended review (2000-2019)	563	Ahmed, M	Emerg. Microbes Infect.	2020
Colistin resistance of Acinetobacter baumannii: clinical reports, mechanisms and antimicrobial strategies	435	Cai, Yun	J. Antimicrob. Chemother.	2012
The rationale for revising the Clinical and Laboratory Standards Institute vancomycin minimal inhibitory concentration interpretive criteria for Staphylococcus aureus	398	Tenover, F	Clin. Infect. Dis.	2007
Guidelines for the control and prevention of meticillin-resistant Staphylococcus aureus (MRSA) in healthcare facilities	367	Coia, J. E.	J. Hosp. Infect.	2006
Antimicrobial Heteroresistance: an Emerging Field in Need of Clarity	332	El-Halfawy, OM.	Clin. Microbiol. Rev.	2015
Helicobacter pylori infection and antibiotic resistance - from biology to clinical implications	318	Tshibangu-Kabamba, E	Nat. Rev. Gastroenterol. Hepatol.	2021
Pseudomonas aeruginosa adaptation and evolution in patients with cystic fibrosis	308	Rossi, Elio	Nat. Rev. Microbiol.	2021
Mechanisms and clinical relevance of bacterial heteroresistance	307	Andersson, Dan I.	Nat. Rev. Microbiol.	2019

Supplementary Table S6. Summary of reported heteroresistance in microorganism-antimicrobial combinations.

Microoganisms	Antimicrobial drugs
Bacillus cereus	meropenem,imipenem(1)
Clostridium difficile	metronidazole(2)
Corynebacterium striatum	daptomycin(3)
Enterococcus faecalis	cefotaxime(4),<br>vancomycin(5),<br>eravacycline(6), omadacycline(7), tigecycline(8)
Enterococcus faecium	vancomycin(9-13),linezolid(11), daptomycin(11),<br>teicoplanin(14), eravacycline(15)
Staphylococcus aureus	beta-lactams(16),oxacillin(17),ceftaroline (18),<br>vancomycin(19, 20), telcoplanin(20), daptomycin(21, 22),<br>trimethoprim-sulfamethoxazole(23),<br>eravacycline(24), omadacycline(25),<br>mupirocin(26),<br>ciprofloxacin(27) ,moxifloxacin(28),<br>gentamicin(29)
Staphylococcus epidermidis	vancomycin(30, 31)
Staphylococcus capitis	vancomycin (32, 33)
Staphylococcus haemolyticus	vancomycin(30, 34, 35)
Staphylococcus hominis	vancomycin(35)
Staphylococcus simulans	vancomycin(35)
Staphylococcus warneri	vancomycin(35)
Streptococcus pneumoniae	telithromycin(36), erythromycin(37)<br>penicillin(38, 39), cephalexin(40),<br>fosfomycin(41)
Streptococcus agalactiae	tigecycline(42)
Achromobacter spp.	polymyxin B(43)
Acinetobacter baumannii	colistin(44), polymyxin B(45),<br>meropenem(46), imipenem(47), doripenem(48),<br>ampicillin-sulbactam(48, 49), ticarcillin-clavulanic acid(49),<br>cefepime(48, 49), cefpirome(49), cefiderocol(50),<br>amikacin(48), gentamicin(48), tobramycin(48, 51),<br>netilmicin(48),<br>trimethoprim/sulfamethoxazole(48),<br>tigecycline(52), eravacycline(53)
Bacteroides fragilis	imipenem(54)
Bordetella bronchiseptica	gentamicin(55)
Burkholderia cenocepacia	polymyxin B(56)
Campylobacter jejuni	azithromycin(57)
Enterobacter cloacae complex	colistin(58),<br>tigecycline(59),

	imipenem(60), meropenem(60),
	ceftazidime/avibactam(61),
	imipenem/relebactam(61),, meropenem/vaborbactam(61),
	fosfomycin(62) ,
	gentamicin(63)
Enterobacter bugandensis	polymyxin B(64)
Enterobacter xiangfangensis	colistin(65)
Escherichia coli	ampicillin(66), piperacillin(67), temocillin(68) ,
	aztreonam(48)
	ceftriaxone(69), cefepime(70), cefotaxime(71),
	ceftazidime(48), cefiderocol(72),
	imipenem(73, 74),meropenem(73, 74),ertapenem(73, 74),
	piperacillin-tazobactam(75, 76),
	ceftolozane/tazobactam(74),ceftazidime/avibactam(61),
	imipenem/relebactam(61), meropenem/vaborbactam(61),
	colistin(77, 78), polymyxin B(79)
	moxifloxacin(28), ciprofloxacin(67, 80),nalidixic acid(67)
	gentamicin(48, 81),tobramycin(82), amikacin(48),
	netilmicin(48),
	fosfomycin(83-85),
	nitrofurantoin(86)
	tetracycline(82),
	chloramphenicol(87),
	azithromycin(87)
Haemophilus influenzae	streptomycin(88), imipenem(89-92)
Helicobacter pylori	clarithromycin(93-95),
	metronidazole(93, 96, 97),
	tetracycline(93, 98, 99),
	furazolidone(93),
	nitrofurantoin(93),
	amoxicillin(100),
	levofloxacin(101), sitafloxacin(102)
Klebsiella pneumoniae	colistin(103), polymyxin B(104)
	meropenem(105), imipenem(106), ertapenem(48),
	aztreonam(48), ampicillin(107),
	cefazolin(107), ceftazidime(107),cefpirome(48),
	cefiderocol(108),
	amoxicillin/clavulanate(107), piperacillin/tazobactam(109),
	cefoperazone/sulbactam(107), ceftazidime/avibactam(61, 110),
	imipenem/relebactam(61), meropenem/vaborbactam(61),

	amikacin(48), tobramycin(48), gentamicin(48), netilmicin(48), trimethoprim/sulfamethoxazole(48), tetracycline(48), eravacycline(111), fosfomycin(83, 112), ciprofloxacin (107), chlorhexidin(113)
Klebsiella aerogenes	carbapenems(114), fosfomycin(62)
Neisseria meningitidis	polymyxin B(115)
Providencia rettgeri	ertapenem(116),meropenem(116)
Pseudomonas aeruginosa	imipenem(117), meropenem(117), colistin(118), polymyxin B(119), cefepime(120), ceftazidime(121), cefiderocol(122, 123) piperacillin/tazobactam(124), ceftolozane/tazobactam(125, 126), ciprofloxacin(121), levofloxacin(127), amikacin(121) , fosfomycin(128), sulfamethoxazole-trimethoprim(129)
Salmonella enterica	colistin(130), tigecycline(48, 131), tetracycline(48), ciprofloxacin(132), fosfomycin(133) ampicillin(133), amoxicillin/clavulanate(133), cephalothin(48), cefepime(48) amikacin(48), tobramycin(48), gentamicin(48), netilmicin(48)
Stenotrophomonas maltophilia	colistin(134)
Mycobacterium avium	clarithromycin(135)
Mycobacterium tuberculosis	rifampin(136),isoniazid(136, 137), streptomycin(137), ethambutol(137), fluoroquinolones(138),aminoglycosides(139), bedaquiline(140, 141),pyrazinamide(142)
Aspergillus fumigatus	itraconazole(143), posaconazole(143), voriconazole(144)
Candida albicans	fluconazole(145-147), itraconazole(145)
Candida glabrata	fluconazole(145, 148, 149), itraconazole(145), amphotericin B(148), caspofungin(150)
Candida krusei	fluconazole(145), itraconazole(145)
Candida parapsilosis	micafungin(151)
Candida tropicalis	fluconazole(148),amphotericin B(148),
Cryptococcus gattii	fluconazole(152), itraconazole(153)
Cryptococcus neoformans	fluconazole(154-156),voriconazole(154)
Trichosporon asahii	voriconazole(157)
Cyanobacteria	aminoglycosides,amphenicols,beta-lactams,macrolides, quinolones, sulfonamides, tetracyclines(158)

Supplementary Figures

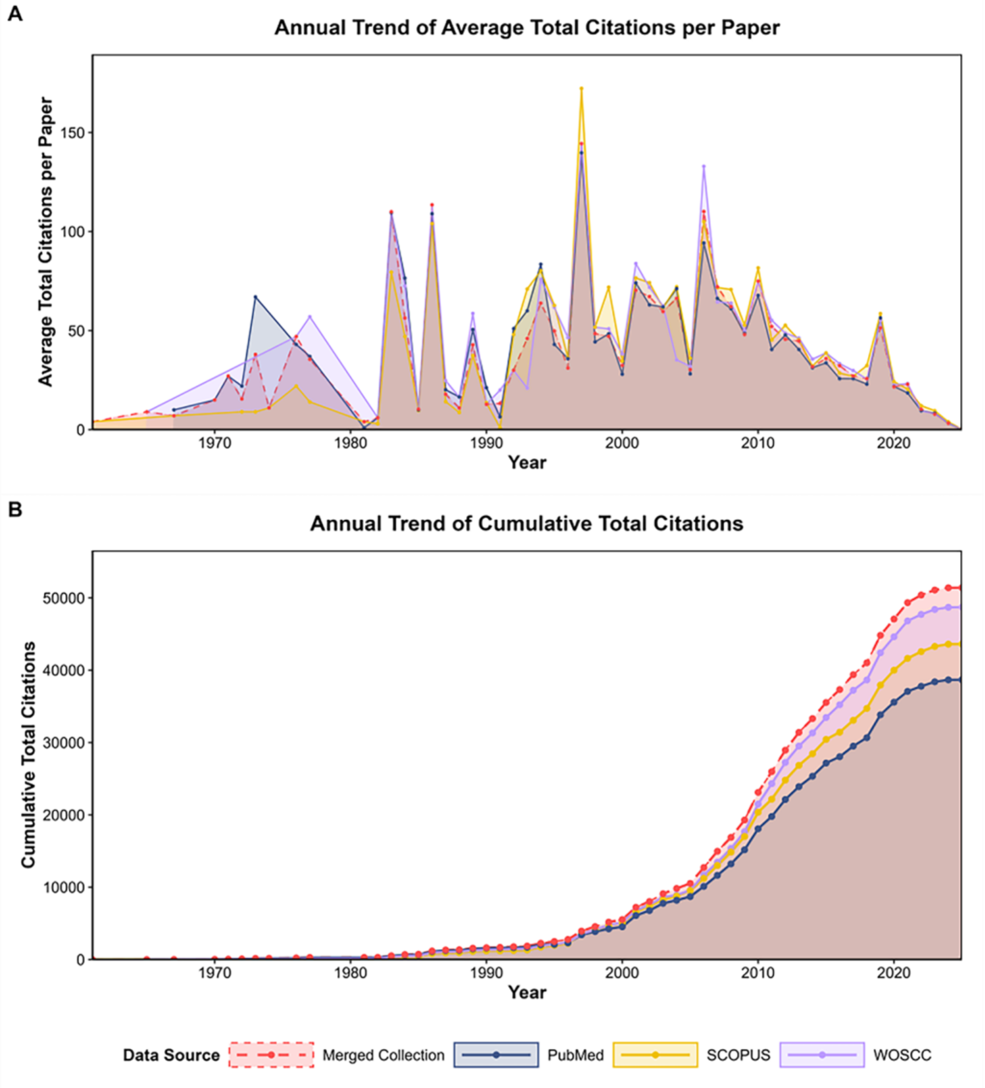

Supplementary Figure S1. Annual Analysis of citations on antibiotic heteroresistance across multiple databases. Annual trend of average total citations per paper(A) and cumulative total citations(B).

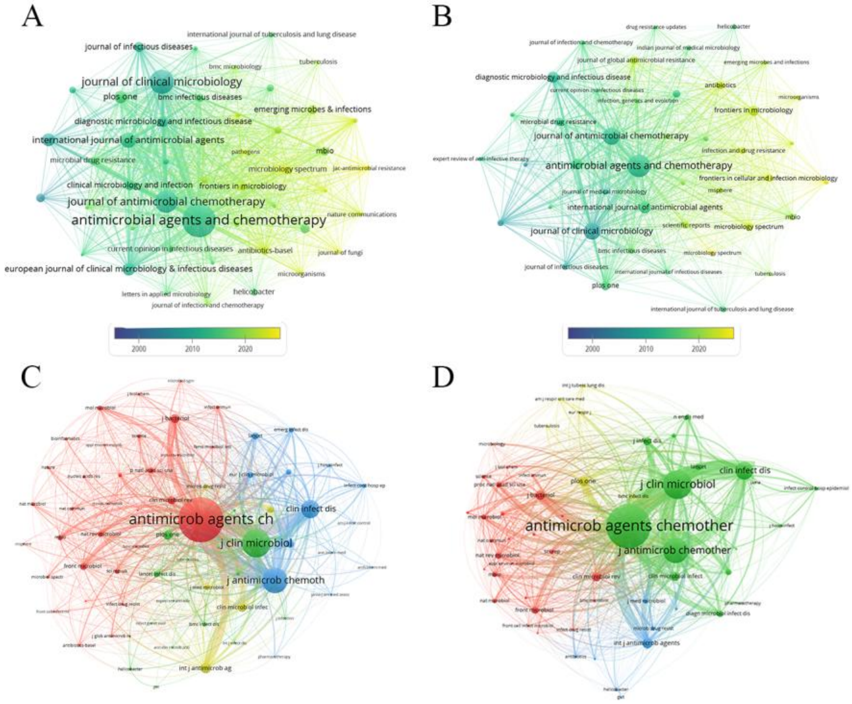

Supplementary Figure S2. Journal analysis of antibiotic heteroresistance in WOSCC and Scopus database. Journal bibliographic coupling analysis of WOSCC (A) and Scopus (B) database were performed by VOSViewer at default settings. Journal co-citation analysis of WOSCC (C) and Scopus (D) database were performed by VOSViewer with minimum citations numbers of one hundred.

Reference

1. Savini V, Favaro M, Fontana C, Catavitello C, Balbinot A, Talia M, et al. Bacillus cereus heteroresistance to carbapenems in a cancer patient. Journal of Hospital Infection. 2009;71(3):288-90.
2. Peláez T, Cercenado E, Alcalá L, Marín M, Martín-López A, Martínez-Alarcón J, et al. Metronidazole resistance in Clostridium difficile is heterogeneous. Journal of Clinical Microbiology. 2008;46(9):3028-32.
3. Tran TT, Jaijakul S, Lewis CT, Diaz L, Panesso D, Kaplan HB, et al. Native Valve Endocarditis Caused by Corynebacterium striatum with Heterogeneous High-Level Daptomycin Resistance: Collateral Damage from Daptomycin Therapy? Antimicrobial Agents and Chemotherapy. 2012;56(6):3461-4.
4. Mainardi JL, Billot-Klein D, Coutrot A, Legrand R, Schoot B, Gutmann L. Resistance to cefotaxime and peptidoglycan composition in Enterococcus faecalis are influenced by exogenous sodium chloride. Microbiology-(UK). 1998;144:2679-85.
5. Cárdenas AM, Andreacchio KA, Edelstein PH. Prevalence and Detection of Mixed-Population Enterococcal Bacteremia. Journal of Clinical Microbiology. 2014;52(7):2604-8.
6. Wen ZW, Shang YP, Xu GJ, Pu ZY, Lin ZW, Bai B, et al. Mechanism of Eravacycline Resistance in Clinical Enterococcus faecalis Isolates From China. Frontiers in Microbiology. 2020;11:10.
7. Lin ZW, Pu ZY, Xu GJ, Bai B, Chen Z, Sun X, et al. Omadacycline Efficacy against Enterococcus faecalis Isolated in China: In Vitro Activity, Heteroresistance, and Resistance Mechanisms. Antimicrobial Agents and Chemotherapy. 2020;64(3):14.
8. Bai B, Chen CC, Zhao YX, Xu GJ, Yu ZJ, Tam VH, et al. In vitro activity of tigecycline and proteomic analysis of tigecycline adaptation strategies in clinical Enterococcus faecalis isolates from China. Journal of Global Antimicrobial Resistance. 2022;30:66-74.
9. Alam MR, Donabedian S, Brown W, Gordon J, Chow JW, Zervos MJ, et al. Heteroresistance to vancomycin in Enterococcus faecium. Journal of Clinical Microbiology. 2001;39(9):3379-81.
10. Khan SA, Sung K, Layton S, Nawaz MS. Heteroresistance to vancomycin and novel point mutations in. Tn1546 of Enterococcus faecium ATCC 51559. International Journal of Antimicrobial Agents. 2008;31(1):27-36.
11. Chacko KI, Sullivan MJ, Beckford C, Altman DR, Ciferri B, Pak TR, et al. Genetic Basis of Emerging Vancomycin, Linezolid, and Daptomycin Heteroresistance in a Case of Persistent Enterococcus faecium Bacteremia. Antimicrobial Agents and Chemotherapy. 2018;62(4):9.
12. Zhou Y, Yang Y, Ding L, Chen CH, Xu XG, Wang MG. Vancomycin Heteroresistance in vanM-type Enterococcus faecium. Microbial Drug Resistance. 2020;26(7):776-82.
13. Sun LY, Zhuang HM, Chen MZ, Chen Y, Chen YY, Shi KR, et al. Vancomycin heteroresistance caused by unstable tandem amplifications of the vanM gene cluster on linear conjugative plasmids in a clinical Enterococcus faecium. Antimicrobial Agents and Chemotherapy. 2024;68(5):15.
14. Qu TT, Zhang JL, Zhou ZH, Wei ZQ, Yu YS, Chen YG, et al. Heteroresistance to Teicoplanin in Enterococcus faecium Harboring the vanA Gene. Journal of Clinical Microbiology. 2009;47(12):4194-6.
15. Wen ZW, Liu FF, Zhang PX, Wei Y, Shi YY, Zheng JX, et al. In vitro activity and adaptation strategies of eravacycline in clinical Enterococcus faecium isolates from China. J Antibiot. 2022;75(9):498-508.

16. Kim C, Mwangi M, Chung M, Milheiriço C, de Lencastre H, Tomasz A. The Mechanism of Heterogeneous Beta-Lactam Resistance in MRSA: Key Role of the Stringent Stress Response. Plos One. 2013;8(12).
17. Liu RS, Zhang J, Du XL, Lv YY, Gao XY, Wang YY, et al. Clonal Diversity, Low-Level and Heterogeneous Oxacillin Resistance of Oxacillin Sensitive MRSA. Infect Drug Resistance. 2021;14:661-9.
18. Saravolatz SN, Martin H, Pawlak J, Johnson LB, Saravolatz LD. Ceftaroline-Heteroresistant Staphylococcus aureus. Antimicrobial Agents and Chemotherapy. 2014;58(6):3133-6.
19. Hiramatsu K, Aritaka N, Hanaki H, Kawasaki S, Hosoda Y, Hori S, et al. Dissemination in Japanese hospitals of strains of Staphylococcus aureus heterogeneously resistant to vancomycin. Lancet (London, England). 1997;350(9092):1670-3.
20. Rybak MJ, Cha R, Cheung CM, Meka VG, Kaatz GW. Clinical isolates of Staphylococcus aureus from 1987 and 1989 demonstrating heterogeneous resistance to vancomycin and telcoplanin. Diagnostic Microbiology and Infectious Disease. 2005;51(2):119-25.
21. Sakoulas G, Alder J, Thauvin-Eliopouios C, Moellering RC, Eliopoulos GM. Induction of daptomycin heterogeneous susceptibility in Staphylococcus aureus by exposure to vancomycin. Antimicrobial Agents and Chemotherapy. 2006;50(4):1581-5.
22. Ji SJ, Jiang SN, Wei X, Sun L, Wang HP, Zhao F, et al. In-Host Evolution of Daptomycin Resistance and Heteroresistance in Methicillin-Resistant Staphylococcus aureus Strains From Three Endocarditis Patients. Journal of Infectious Diseases. 2020;221:S243-S52.
23. Coelho C, de Lencastre H, Aires-de-Sousa M. Frequent occurrence of trimethoprim-sulfamethoxazole hetero-resistant Staphylococcus aureus isolates in different African countries. European Journal of Clinical Microbiology & Infectious Diseases. 2017;36(7):1243-52.
24. Zhang F, Bai B, Xu GJ, Lin ZW, Li GQ, Chen Z, et al. Eravacycline activity against clinical S-aureus isolates from China: in vitro activity, MLST profiles and heteroresistance. BMC Microbiol. 2018;18:9.
25. Bai B, Lin ZW, Pu ZY, Xu GJ, Zhang F, Chen Z, et al. In vitro Activity and Heteroresistance of Omadacycline Against Clinical Staphylococcus aureus Isolates From China Reveal the Impact of Omadacycline Susceptibility by Branched-Chain Amino Acid Transport System II Carrier Protein, Na/Pi Cotransporter Family Protein, and Fibronectin-Binding Protein. Frontiers in Microbiology. 2019;10:12.
26. Mutu E, Chen GL, Liu RS, Wang YJ. High prevalence of heterogeneous mupirocin-resistant Staphylococcus aureus and its molecular characterization. Am J Transl Res. 2022;14(11):8243-+.
27. Li MY, Jian QT, Ye XY, Jing M, Wu JE, Wu ZH, et al. Mechanisms of mepA Overexpression and Membrane Potential Reduction Leading to Ciprofloxacin Heteroresistance in a Staphylococcus aureus Isolate. Int J Mol Sci. 2025;26(5).
28. Iqbal K, Broeker A, Nowak H, Rahmel T, Nussbaumer-Pröll A, Österreicher Z, et al. A pharmacometric approach to define target site-specific breakpoints for bacterial killing and resistance suppression integrating microdialysis, time-kill curves and heteroresistance data: a case study with moxifloxacin. Clinical Microbiology and Infection. 2020;26(9):8.
29. Heidarian S, Guliaev A, Nicoloff H, Hjort K, Andersson DI. High prevalence of heteroresistance in Staphylococcus aureus is caused by a multitude of mutations in core genes. PLoS Biol. 2024;22(1):24.
30. Nunes APF, Teixeira LM, Iorio NLP, Bastos CCR, Fonseca LD, Souto-Padrón T, et al. Heterogeneous resistance to vancomycin in Staphylococcus epidermidis, Staphylococcus haemolyticus and Staphylococcus warneri clinical strains:: characterisation of glycopeptide

susceptibility profiles and cell wall thickening. International Journal of Antimicrobial Agents. 2006;27(4):307-15.
31. Chong J, Quach C, Blanchard AC, Poliquin PG, Golding GR, Laferrière C, et al. Molecular Epidemiology of a Vancomycin-Intermediate Heteroresistant Staphylococcus epidermidis Outbreak in a Neonatal Intensive Care Unit. Antimicrobial Agents and Chemotherapy. 2016;60(10):5673-81.
32. Van Der Zwet WC, Debets-Ossenkopp YJ, Reinders E, Kapi M, Savelkoul PHM, Van Elburg RM, et al. Nosocomial spread of a Staphylococcus capitis strain with heteroresistance to vancomycin in a neonatal intensive care unit. Journal of Clinical Microbiology. 2002;40(7):2520-5.
33. D'Mello D, Daley AJ, Rahman MS, Qu Y, Garland S, Pearce C, et al. Vancomycin heteroresistance in bloodstream isolates of Staphylococcus capitis. Journal of Clinical Microbiology. 2008;46(9):3124-6.
34. Bathavatchalam YD, Solaimalai D, Amladi A, Dwarakanathan HT, Anandan S, Veeraraghavan B. Vancomycin heteroresistance in Staphylococcus haemolyticus: elusive phenotype. Futur Sci OA. 2021;7(7):7.
35. Szemraj M, Lisiecki P, Glajzner P, Szewczyk EM. Vancomycin heteroresistance among methicillin-resistant clinical isolates S. haemolyticus, S. hominis, S. simulans, and S. warneri. Brazilian Journal of Microbiology. 2023;54(1):159-67.
36. Rantala M, Haanpera-Heikkinen M, Lindgren M, Seppälä H, Huovinen P, Jalava J. Streptococcus pneumoniae isolates resistant to telithromycin. Antimicrobial Agents and Chemotherapy. 2006;50(5):1855-8.
37. Lohsen S, Stephens DS. Inducible Mega-Mediated Macrolide Resistance Confers Heteroresistance in Streptococcus pneumoniae. Antimicrobial Agents and Chemotherapy. 2023;67(3):12.
38. Morand B, Mühlemann K. Heteroresistance to penicillin in Streptococcus pneumoniae. Proceedings of the National Academy of Sciences of the United States of America. 2007;104(35):14098-103.
39. Engel H, Mika M, Denapaite D, Hakenbeck R, Mühlemann K, Heller M, et al. A Low-Affinity Penicillin-Binding Protein 2x Variant Is Required for Heteroresistance in Streptococcus pneumoniae. Antimicrobial Agents and Chemotherapy. 2014;58(7):3934-41.
40. Sorg RA, Veening JW. Microscale insights into pneumococcal antibiotic mutant selection windows. Nature Communications. 2015;6:13.
41. Engel H, Gutiérrez-Fernández J, Flückiger C, Martínez-Ripoll M, Mühlemann K, Hermoso JA, et al. Heteroresistance to Fosfomycin Is Predominant in Streptococcus pneumoniae and Depends on the murA1 Gene. Antimicrobial Agents and Chemotherapy. 2013;57(6):2801-8.
42. Li PY, Wei Y, Li GQ, Cheng H, Xu ZC, Yu ZJ, et al. Comparison of antimicrobial efficacy of eravacycline and tigecycline against clinical isolates of Streptococcus agalactiae in China: In vitro activity, heteroresistance, and cross-resistance. Microb Pathog. 2020;149:7.
43. MacDonald L, Keenan S, Di Lorenzo F, Adade NE, Kenna DTD, Millar BC, et al. Polymyxin Resistance and Heteroresistance Are Common in Clinical Isolates of Achromobacter Species and Correlate with Modifications of the Lipid A Moiety of Lipopolysaccharide. Microbiol Spectr. 2023;11(1):19.
44. Li J, Rayner CR, Nation RL, Owen RJ, Spelman D, Tan KE, et al. Heteroresistance to colistin in multidrug-resistant Acinetobacter baumannii. Antimicrobial Agents and Chemotherapy. 2006;50(9):2946-50.

45. Barin J, Martins AF, Heineck BL, Barth AL, Zavascki AP. Hetero- and adaptive resistance to polymyxin B in OXA-23-producing carbapenem-resistant Acinetobacter baumannii isolates. Annals of Clinical Microbiology and Antimicrobials. 2013;12:5.
46. Ikonomidis A, Neou E, Gogou V, Vrioni G, Tsakris A, Pournaras S. Heteroresistance to Meropenem in Carbapenem-Susceptible Acinetobacter baumannii. Journal of Clinical Microbiology. 2009;47(12):4055-9.
47. Lee HY, Chen CL, Wang SB, Su LH, Chen SH, Liu SY, et al. Imipenem heteroresistance induced by imipenem in multidrug-resistant Acinetobacter baumannii: mechanism and clinical implications. International Journal of Antimicrobial Agents. 2011;37(4):302-8.
48. Nicoloff H, Hjort K, Levin BR, Andersson DI. The high prevalence of antibiotic heteroresistance in pathogenic bacteria is mainly caused by gene amplification. Nature Microbiology. 2019;4(3):504-14.
49. Hung KH, Wang MC, Huang AH, Yan JJ, Wu JJ. Heteroresistance to Cephalosporins and Penicillins in Acinetobacter baumannii. Journal of Clinical Microbiology. 2012;50(3):721-6.
50. Stracquadanio S, Bonomo C, Marino A, Bongiorno D, Privitera GF, Bivona DA, et al. Acinetobacter baumannii and Cefiderocol, between Cidality and Adaptability. Microbiol Spectr. 2022;10(5):11.
51. Anderson SE, Sherman EX, Weiss DS, Rather PN. Aminoglycoside Heteroresistance in Acinetobacter baumannii AB5075. Msphere. 2018;3(4):12.
52. Jo J, Ko KS. Tigecycline Heteroresistance and Resistance Mechanism in Clinical Isolates of Acinetobacter baumannii. Microbiol Spectr. 2021;9(2):9.
53. Li YT, Chen XD, Guo YY, Lin SW, Wang MZ, Xu JB, et al. Emergence of eravacycline heteroresistance in carbapenem-resistant Acinetobacter baumannii isolates in China. Frontiers in Cellular and Infection Microbiology. 2024;14:13.
54. Baaity Z, von Loewenich FD, Nagy E, Orosz L, Burián K, Somogyvári F, et al. Phenotypic and Molecular Characterization of Carbapenem-Heteroresistant Bacteroides fragilis Strains. Antibiotics-Basel. 2022;11(5):15.
55. Yi L, Fan HR, Yuan S, Li RS, Wang HK, Quan YY, et al. Antimicrobial Resistance and Biofilm Formation of Bordetella bronchiseptica in Central China, with Evidence of a Rare Heteroresistance Strain to Gentamicin. Animals. 2024;14(9):13.
56. El-Halfawy OM, Valvano MA. Chemical Communication of Antibiotic Resistance by a Highly Resistant Subpopulation of Bacterial Cells. Plos One. 2013;8(7):10.
57. Lurchachaiwong W, Ruksasiri S, Wassanarungroj P, Serichantalergs O, Bodhidatta L, Crawford J, et al. Determination of azithromycin heteroresistant Campylobacter jejuni in traveler's diarrhea. Gut Pathogens. 2019;11:5.
58. Band VI, Crispell EK, Napier BA, Herrera CM, Tharp GK, Vavikolanu K, et al. Antibiotic failure mediated by a resistant subpopulation in Enterobacter cloacae. Nature Microbiology. 2016;1(6):9.
59. Liu H, Jia XJ, Zou H, Sun S, Li S, Wang YH, et al. Detection and characterization of tigecycline heteroresistance in E. cloacae: clinical and microbiological findings. Emerg Microbes Infect. 2019;8(1):564-74.
60. da Silva AEB, Martins AF, Nodari CS, Magagnin CM, Barth AL. Carbapenem-heteroresistance among isolates of the Enterobacter cloacae complex: is it a real concern? European Journal of Clinical Microbiology & Infectious Diseases. 2018;37(1):185-6.
61. Lin CK, Page A, Lohsen S, Haider AA, Waggoner J, Smith G, et al. Rates of resistance and heteroresistance to newer β-lactam/β-lactamase inhibitors for carbapenem-resistant Enterobacterales. JAC-Antimicrob Resist. 2024;6(2):7.

62. Lim TP, Teo JQM, Goh AWL, Tan SX, Koh TH, Lee WHL, et al. In Vitro Pharmacodynamics of Fosfomycin against Carbapenem-Resistant Enterobacter cloacae and Klebsiella aerogenes. Antimicrobial Agents and Chemotherapy. 2020;64(9):9.
63. Choi AJ, Bennison DJ, Kulkarni E, Azar H, Sun HY, Li HQ, et al. Aminoglycoside heteroresistance in Enterobacter cloacae is driven by the cell envelope stress response. Mbio. 2024;15(12).
64. García-Romero I, Srivastava M, Monjarás-Feria J, Korankye SO, MacDonald L, Scott NE, et al. Drug efflux and lipid A modification by 4-L-aminoarabinose are key mechanisms of polymyxin B resistance in the sepsis pathogen Enterobacter bugandensis. Journal of Global Antimicrobial Resistance. 2024;37:108-21.
65. Yao YC, Doijad S, Falgenhauer J, Schmiedel J, Imirzalioglu C, Chakraborty T. Co-occurrence of dual carbapenemases KPC-2 and OXA-48 with the mobile colistin resistance gene mcr-9.1 in Enterobacter xiangfangensis. Frontiers in Cellular and Infection Microbiology. 2022;12:11.
66. Dai YC, Li CY, Yi J, Qin Q, Liu BH, Qiao L. Plasmonic Colloidosome-Coupled MALDI-TOF MS for Bacterial Heteroresistance Study at Single-Cell Level. Analytical Chemistry. 2020;92(12):8051-7.
67. Diaz-Diaz S, Yerbes P, Recacha E, de Gregorio-Iaria B, Pulido MR, Romero-Munoz M, et al. RecA inactivation as a strategy to reverse the heteroresistance phenomenon in clinical isolates of Escherichia coli. International Journal of Antimicrobial Agents. 2023;61(2):9.
68. Mallart E, Guerin F, Amoura A, Le Scouarnec M, Hamon A, El Meouche I, et al. Impact of the phenotypic expression of temocillin resistance in Escherichia coli on temocillin efficacy in a murine peritonitis model. Journal of Antimicrobial Chemotherapy. 2024;79(5):1051-9.
69. Wang XR, Kang Y, Luo CX, Zhao T, Liu L, Jiang XD, et al. Heteroresistance at the Single-Cell Level: Adapting to Antibiotic Stress through a Population-Based Strategy and Growth-Controlled Interphenotypic Coordination. Mbio. 2014;5(1):9.
70. Ma W, Sun J, Yang S, Zhang L. Epidemiological and clinical features for cefepime heteroresistant Escherichia coli infections in Southwest China. European Journal of Clinical Microbiology & Infectious Diseases. 2016;35(4):571-8.
71. Scheler O, Makuch K, Debski PR, Horka M, Ruszczak A, Pacocha N, et al. Droplet-based digital antibiotic susceptibility screen reveals single-cell clonal heteroresistance in an isogenic bacterial population. Scientific Reports. 2020;10(1):8.
72. Liu HW, Zhou P, Ma P, Liu YQ, Zhang YF, Li QW, et al. Carbapenem-resistant Gram-negative bacteria exhibiting clinically undetected cefiderocol heteroresistance leads to treatment failure in a murine model of infection. Frontiers in Microbiology. 2025;16.
73. Sun JD, Huang SF, Yang SS, Pu SL, Zhang CM, Zhang LP. Impact of carbapenem heteroresistance among clinical isolates of invasive Escherichia coli in Chongqing, southwestern China. Clinical Microbiology and Infection. 2015;21(5):10.
74. Tan KR, Nguyen J, Nguyen K, Huse HK, Nieberg PH, Wong-Beringer A. Prevalence of the carbapenem-heteroresistant phenotype among ESBL-producing Escherichia coli and Klebsiella pneumoniae clinical isolates. Journal of Antimicrobial Chemotherapy. 2020;75(6):1506-12.
75. Shubert C, Slaughter J, Creely D, van Belkum A, Gayral JP, Dunne WM, et al. Population Analysis of Escherichia coli Isolates with Discordant Resistance Levels by Piperacillin-Tazobactam Broth Microdilution and Agar Dilution Testing. Antimicrobial Agents and Chemotherapy. 2014;58(3):1779-81.

76. Rodríguez-Villodres A, de la Rosa JMO, Alvarez-Marín R, Pachón J, Aznar J, Lepe JA, et al. Heteroresistance to Piperacillin-Tazobactam in Clinical Isolates of Escherichia coli Sequence Type 131. Antimicrobial Agents and Chemotherapy. 2018;62(1):3.
77. Kuang QH, He DD, Sun HR, Hu HH, Li FL, Li WY, et al. R93P Substitution in the PmrB HAMP Domain Contributes to Colistin Heteroresistance in Escherichia coli Isolates from Swine. Antimicrobial Agents and Chemotherapy. 2020;64(11):9.
78. Liao WL, Lin J, Jia HY, Zhou C, Zhang Y, Lin YS, et al. Resistance and Heteroresistance to Colistin in Escherichia coli Isolates from Wenzhou, China. Infect Drug Resistance. 2020;13:3551-61.
79. Smith NM, Chan AR, Nguyen TD, Dumbleton JT. Bacterial Mixology: Combining Pharmacodynamic Models to Predict In Vitro Competition of MCR-1-Harboring E. coli. Antibiotics-Basel. 2022;11(1):8.
80. Bauer D, Wieland K, Qiu L, Neumann-Cip AC, Magistro G, Stief C, et al. Heteroresistant Bacteria Detected by an Extended Raman-Based Antibiotic Susceptibility Test. Analytical Chemistry. 2020;92(13):8722-31.
81. Heyman G, Jonsson S, Fatsis-Kavalopoulos N, Hjort K, Nicoloff H, Furebring M, et al. Prevalence, misclassification, and clinical consequences of the heteroresistant phenotype in Escherichia coli bloodstream infections in patients in Uppsala, Sweden: a retrospective cohort study. Lancet Microbe. 2025;6(4).
82. Pereira C, Larsson J, Hjort K, Elf J, Andersson DI. The highly dynamic nature of bacterial heteroresistance impairs its clinical detection. Commun Biol. 2021;4(1):12.
83. Abbott IJ, van Gorp E, Wijma RA, Meletiadis J, Roberts JA, Mouton JW, et al. Oral Fosfomycin Efficacy with Variable Urinary Exposures following Single and Multiple Doses against Enterobacterales: the Importance of Heteroresistance for Growth Outcome. Antimicrobial Agents and Chemotherapy. 2020;64(3):13.
84. Campos ACD, Andrade NL, Couto N, Mutters NT, de Vos M, Rosa ACD, et al. Characterization of fosfomycin heteroresistance among multidrug-resistant Escherichia coli isolates from hospitalized patients in Rio de Janeiro, Brazil. Journal of Global Antimicrobial Resistance. 2020;22:584-93.
85. Portillo-Calderón I, Ortiz-Padilla M, Rodríguez-Martínez JM, de Gregorio-Iaria B, Blázquez J, Rodríguez-Baño J, et al. Contribution of hypermutation to fosfomycin heteroresistance in Escherichia coli. Journal of Antimicrobial Chemotherapy. 2020;75(8):2066-75.
86. Wan Y, Sabnis A, Mumin Z, Potterill I, Jauneikaite E, Brown CS, et al. IS1-related large- scale deletion of chromosomal regions harbouring the oxygen- insensitive nitroreductase gene nfsB causes nitrofurantoin heteroresistance in Escherichia coli. Microb Genomics. 2023;9(9):11.
87. Gil-Gil T, Berryhill BA, Manuel JA, Smith AP, McCall IC, Baquero F, et al. The evolution of heteroresistance via small colony variants in Escherichia coli following long term exposure to bacteriostatic antibiotics. Nature Communications. 2024;15(1).
88. Alexander HE, Leidy G. MODE OF ACTION OF STREPTOMYCIN ON TYPE b HEMOPHILUS INFLUENZAE : II. NATURE OF RESISTANT VARIANTS. The Journal of experimental medicine. 1947;85(6):607-21.
89. Cerquetti M, Giufrè M, Cardines R, Mastrantonio P. First characterization of heterogeneous resistance to imipenem in invasive nontypeable Haemophilus influenzae isolates. Antimicrobial Agents and Chemotherapy. 2007;51(9):3155-61.
90. Cherkaoui A, Diene SM, Renzoni A, Emonet S, Renzi G, François P, et al. Imipenem heteroresistance in nontypeable Haemophilus influenzae is linked to a combination of altered PBP3, slow drug influx and direct efflux regulation. Clinical Microbiology and Infection. 2017;23(2):11.

91. Cherkaoui A, Gaïa N, Baud D, Leo S, Fischer A, Ruppe E, et al. Molecular characterization of fluoroquinolones, macrolides, and imipenem resistance in Haemophilus influenzae: analysis of the mutations in QRDRs and assessment of the extent of the AcrAB-TolC-mediated resistance. European Journal of Clinical Microbiology & Infectious Diseases. 2018;37(11):2201-10.
92. Gil-Campillo C, González-Díaz A, Rapún-Araiz B, Iriarte-Elizaintzin O, Elizalde-Gutiérrez I, Fernández-Calvet A, et al. Imipenem heteroresistance but not tolerance in Haemophilus influenzae during chronic lung infection associated with chronic obstructive pulmonary disease. Frontiers in Microbiology. 2023;14:19.
93. Kim JJ, Kim JG, Kwon DH. Mixed-infection of antibiotic susceptible and resistant Helicobacter pylori isolates in a single patient and underestimation of antimicrobial susceptibility testing. Helicobacter. 2003;8(3):202-6.
94. Arévalo-Jaimes BV, Rojas-Rengifo DF, Jaramillo CA, de Molano BM, Vera-Chamorro JF, Delgado MD. Genotypic determination of resistance and heteroresistance to clarithromycin in Helicobacter pylori isolates from antrum and corpus of Colombian symptomatic patients. Bmc Infectious Diseases. 2019;19:8.
95. Wang YH, Gong XL, Liu DW, Zeng R, Zhou LF, Sun XY, et al. Characteristics of Helicobacter pylori Heteroresistance in Gastric Biopsies and Its Clinical Relevance. Frontiers in Cellular and Infection Microbiology. 2022;11:7.
96. Lee YC, Lee SY, Pyo JH, Kwon DH, Rhee JC, Kim JJ. Isogenic variation of Helicobacter pylori strain resulting in heteroresistant antibacterial phenotypes in a single host in vivo. Helicobacter. 2005;10(3):240-8.
97. Matteo MJ, Pérez CV, Domingo MR, Olmos M, Sanchez C, Catalano M. DNA sequence analysis of rdxA and frxA from paired metronidazole-sensitive and -resistant Helicobacter pylori isolates obtained from patients with heteroresistance. International Journal of Antimicrobial Agents. 2006;27(2):152-8.
98. Kotilea K, Iliadis E, Nguyen J, Salame A, Mahler T, Deyi VYM, et al. Antibiotic resistance, heteroresistance, and eradication success of Helicobacter pylori infection in children. Helicobacter. 2023;28(5):9.
99. Nguyen TC, Le GKN, Pham DTH, Pham BV, Nguyen LTH, Che TH, et al. Antibiotic resistance and heteroresistance in Helicobacter pylori isolates from symptomatic Vietnamese children: A prospective multicenter study. Helicobacter. 2023;28(5):12.
100. Matteo MJ, Granados G, Olmos M, Wonaga A, Catalano M. Helicobacter pylori amoxicillin heteroresistance due to point mutations in PBP-1A in isogenic isolates. Journal of Antimicrobial Chemotherapy. 2008;61(3):474-7.
101. Kao CY, Lee AY, Huang AH, Song PY, Yang YJ, Sheu SM, et al. Heteroresistance of Helicobacter pylori from the same patient prior to antibiotic treatment. Infection Genetics and Evolution. 2014;23:196-202.
102. Islam JM, Yano Y, Okamoto A, Matsuda R, Shiraishi M, Hashimoto Y, et al. Evidence of Helicobacter pylori heterogeneity in human stomachs by susceptibility testing and characterization of mutations in drug-resistant isolates. Scientific Reports. 2024;14(1):16.
103. Poudyal A, Howden BP, Bell JM, Gao W, Owen RJ, Turnidge JD, et al. In vitro pharmacodynamics of colistin against multidrug-resistant Klebsiella pneumoniae. Journal of Antimicrobial Chemotherapy. 2008;62(6):1311-8.
104. Ma XY, He YT, Yu XG, Cai YM, Zeng JM, Cai RX, et al. Ceftazidime/avibactam Improves the Antibacterial Efficacy of Polymyxin B Against Polymyxin B Heteroresistant KPC-2-Producing

Klebsiella pneumoniae and Hinders Emergence of Resistant Subpopulation in vitro. Frontiers in Microbiology. 2019;10:10.

105. Pournaras S, Kristo I, Vrioni G, Ikonomidis A, Poulou A, Petropoulou D, et al. Characteristics of Meropenem Heteroresistance in Klebsiella pneumoniae Carbapenemase (KPC)-Producing Clinical Isolates of K-pneumoniae. Journal of Clinical Microbiology. 2010;48(7):2601-4.

106. Tato M, Morosini M, García L, Albertí S, Coque MT, Cantón R. Carbapenem Heteroresistance in VIM-1-Producing Klebsiella pneumoniae Isolates Belonging to the Same Clone: Consequences for Routine Susceptibility Testing. Journal of Clinical Microbiology. 2010;48(11):4089-93.

107. Zhang QY, Wen LR, Li SS, Zheng LW, Nie YL, Chen JS. Overview of heteroresistance to multiple antibiotics in clinical Klebsiella pneumoniae isolates and combination therapeutic strategies. JAC-Antimicrob Resist. 2025;7(3).

108. Witt LS, Steed DB, Burd EM, Ozturk T, Davis MH, Satola SW, et al. Bacteraemia with an MBL-producing Klebsiella pneumoniae: treatment and the potential role of cefiderocol heteroresistance. Journal of Antimicrobial Chemotherapy. 2022;77(9):2569-71.

109. Babiker A, Lohsen S, Van Riel J, Hjort K, Weiss DS, Andersson D, et al. Heteroresistance to piperacillin/tazobactam in Klebsiella pneumoniae is mediated by increased copy number of multiple β-lactamase genes. JAC-Antimicrob Resist. 2024;6(2):6.

110. Li YT, Chen XD, Guo YY, Lin YZ, Wang XH, He GH, et al. Overexpression of KPC contributes to ceftazidime-avibactam heteroresistance in clinical isolates of carbapenem-resistant Klebsiella pneumoniae. Frontiers in Cellular and Infection Microbiology. 2024;14.

111. Zheng JX, Lin ZW, Sun X, Lin WH, Chen Z, Wu Y, et al. Overexpression of OqxAB and MacAB efflux pumps contributes to eravacycline resistance and heteroresistance in clinical isolates of Klebsiella pneumoniae. Emerg Microbes Infect. 2018;7:11.

112. Abbott IJ, Dekker J, van Gorp E, Wijma RA, Raaphorst MN, Klaassen CHW, et al. Impact of bacterial species and baseline resistance on fosfomycin efficacy in urinary tract infections. Journal of Antimicrobial Chemotherapy. 2020;75(4):988-96.

113. Naparstek L, Carmeli Y, Chmelnitsky I, Banin E, Navon-Venezia S. Reduced susceptibility to chlorhexidine among extremely-drug-resistant strains of Klebsiella pneumoniae. Journal of Hospital Infection. 2012;81(1):15-9.

114. Gordon NC, Wareham DW. Failure of the MicroScan WalkAway System To Detect Heteroresistance to Carbapenems in a Patient with Enterobacter aerogenes Bacteremia. Journal of Clinical Microbiology. 2009;47(9):3024-5.

115. Tzeng YL, Berman Z, Toh E, Bazan JA, Turner AN, Retchless AC, et al. Heteroresistance to the model antimicrobial peptide polymyxin B in the emerging Neisseria meningitidis lineage 11.2 urethritis clade: mutations in the pilMNOPQ operon. Molecular Microbiology. 2019;111(1):254-68.

116. Zavascki AP, Falci DR, da Silva RCF, Dalarosa MG, Ribeiro VB, Rozales FP, et al. Heteroresistance to Carbapenems in New Delhi Metallo-β-Lactamase-1-Producing Isolates: A Challenge for Detection? Infection Control and Hospital Epidemiology. 2014;35(6):751-2.

117. Pournaras S, Ikonomidis A, Markogiannakis A, Spanakis N, Maniatis AN, Tsakris A. Characterization of clinical isolates of Pseudomonas aeruginosa heterogeneously resistant to carbapenems. Journal of Medical Microbiology. 2007;56(1):66-70.

118. Bergen PJ, Forrest A, Bulitta JB, Tsuji BT, Sidjabat HE, Paterson DL, et al. Clinically Relevant Plasma Concentrations of Colistin in Combination with Imipenem Enhance Pharmacodynamic Activity against Multidrug-Resistant Pseudomonas aeruginosa at Multiple Inocula. Antimicrobial Agents and Chemotherapy. 2011;55(11):5134-42.

119. Hermes DM, Pitt CP, Lutz L, Teixeira AB, Ribeiro VB, Netto B, et al. Evaluation of heteroresistance to polymyxin B among carbapenem-susceptible and -resistant Pseudomonas aeruginosa. Journal of Medical Microbiology. 2013;62:1184-9.
120. Jia XJ, Ma WJ, He JC, Tian XL, Liu H, Zou H, et al. Heteroresistance to cefepime in Pseudomonas aeruginosa bacteraemia. International Journal of Antimicrobial Agents. 2020;55(3):10.
121. Lu Y, Liu YY, Zhou CX, Liu YQ, Long YF, Lin DL, et al. Quorum sensing regulates heteroresistance in Pseudomonas aeruginosa. Frontiers in Microbiology. 2022;13:11.
122. Egge SL, Rizvi SA, Simar SR, Alcalde M, Martinez JRW, Hanson BM, et al. Cefiderocol heteroresistance associated with mutations in TonB-dependent receptor genes in Pseudomonas aeruginosa of clinical origin. Antimicrobial Agents and Chemotherapy. 2024;68(8):16.
123. Teran N, Egge SL, Phe K, Baptista RP, Tam VH, Miller WR, et al. The emergence of cefiderocol resistance in Pseudomonas aeruginosa from a heteroresistant isolate during prolonged therapy. Antimicrobial Agents and Chemotherapy. 2024;68(1):5.
124. Pournaras S, Ikonomidis A, Neou E, Kantzanou M, Maniatis AN, Tsakris A. Piperacillin/tazobactam-heteroresistant Pseudomonas aeruginosa from urinary infection, successfully treated by piperacillin/tazobactam. Journal of Antimicrobial Chemotherapy. 2008;61(3):757-8.
125. Monogue ML, Sanders JM, Pybus CA, Kim J, Zhan XW, Clark AE, et al. Ceftolozane/tazobactam heteroresistance in cystic fibrosis-related Pseudomonas aeruginosa infections. JAC-Antimicrob Resist. 2023;5(4):8.
126. Tait JR, Harper M, Cortés-Lara S, Rogers KE, López-Causapé C, Smallman TR, et al. Ceftolozane-Tazobactam against Pseudomonas aeruginosa Cystic Fibrosis Clinical Isolates in the Hollow-Fiber Infection Model: Challenges Imposed by Hypermutability and Heteroresistance. Antimicrobial Agents and Chemotherapy. 2023;67(8):12.
127. Li WR, Zhang ZQ, Liao K, Wang BB, Liu HZ, Shi QS, et al. Pseudomonas aeruginosa heteroresistance to levofloxacin caused by upregulated expression of essential genes for DNA replication and repair. Frontiers in Microbiology. 2022;13:15.
128. Walsh CC, McIntosh MP, Peleg AY, Kirkpatrick CM, Bergen PJ. In vitro pharmacodynamics of fosfomycin against clinical isolates of Pseudomonas aeruginosa. Journal of Antimicrobial Chemotherapy. 2015;70(11):3042-50.
129. Qin X, Zhou C, Zerr DM, Adler A, Addetia A, Yuan SH, et al. Heterogeneous Antimicrobial Susceptibility Characteristics in Pseudomonas aeruginosa Isolates from Cystic Fibrosis Patients. Msphere. 2018;3(2):17.
130. Hjort K, Nicoloff H, Andersson DI. Unstable tandem gene amplification generates heteroresistance (variation in resistance within a population) to colistin in Salmonella enterica. Molecular Microbiology. 2016;102(2):274-89.
131. Chen Y, Hu DX, Zhang QJ, Liao XP, Liu YH, Sun J. Efflux Pump Overexpression Contributes to Tigecycline Heteroresistance in Salmonella enterica serovar Typhimurium. Frontiers in Cellular and Infection Microbiology. 2017;7:8.
132. Zhang CZ, Zhang Y, Ding XM, Lin XL, Lian XL, Trampari E, et al. Emergence of ciprofloxacin heteroresistance in foodborne Salmonella enterica serovar Agona. Journal of Antimicrobial Chemotherapy. 2020;75(10):2773-9.
133. Zwe YH, Chin SF, Kohli GS, Aung KT, Yang L, Yuk HG. Whole genome sequencing (WGS) fails to detect antimicrobial resistance (AMR) from heteroresistant subpopulation of Salmonella enterica. Food Microbiol. 2020;91:7.

134. Martinez-Servat S, Yero D, Huedo P, Marquez R, Molina G, Daura X, et al. Heterogeneous Colistin-Resistance Phenotypes Coexisting in Stenotrophomonas maltophilia Isolates Influence Colistin Susceptibility Testing. Frontiers in Microbiology. 2018;9:10.
135. Ito A, Nanjo Y, Kajiwara C, Shiozawa A, Urabe N, Homma S, et al. Intrinsic clarithromycin heteroresistance in Mycobacterium avium. Journal of Infection and Chemotherapy. 2024;30(8):752-6.
136. Hofmann-Thiel S, van Ingen J, Feldmann K, Turaev L, Uzakova GT, Murmusaeva G, et al. Mechanisms of heteroresistance to isoniazid and rifampin of Mycobacterium tuberculosis in Tashkent, Uzbekistan. European Respiratory Journal. 2009;33(2):368-74.
137. Rinder H, Mieskes KT, Löscher T. Heteroresistance in Mycobacterium tuberculosis. International Journal of Tuberculosis and Lung Disease. 2001;5(4):339-45.
138. Zhang XB, Zhao B, Liu LG, Zhu YF, Zhao YL, Jin Q. Subpopulation Analysis of Heteroresistance to Fluoroquinolone in Mycobacterium tuberculosis Isolates from Beijing, China. Journal of Clinical Microbiology. 2012;50(4):1471-4.
139. Pholwat S, Stroup S, Foongladda S, Houpt E. Digital PCR to Detect and Quantify Heteroresistance in Drug Resistant Mycobacterium tuberculosis. Plos One. 2013;8(2):10.
140. Nimmo C, Brien K, Millard J, Grant AD, Padayatchi N, Pym AS, et al. Dynamics of within-host Mycobacterium tuberculosis diversity and heteroresistance during treatment. EBioMedicine. 2020;55:13.
141. Madadi-Goli N, Ahmadi K, Kamakoli MK, Azizi M, Khanipour S, Dizaji SP, et al. The importance of heteroresistance and efflux pumps in bedaquiline-resistant Mycobacterium tuberculosis isolates from Iran. Annals of Clinical Microbiology and Antimicrobials. 2024;23(1):10.
142. Werngren J, Mansjö M, Glader M, Hoffner S, Forsman LD. Detection of Pyrazinamide Heteroresistance in Mycobacterium tuberculosis. Antimicrobial Agents and Chemotherapy. 2021;65(9):9.
143. Escribano P, Recio S, Peláez T, González-Rivera M, Bouza E, Guinea J. In Vitro Acquisition of Secondary Azole Resistance in Aspergillus fumigatus Isolates after Prolonged Exposure to Itraconazole: Presence of Heteroresistant Populations. Antimicrobial Agents and Chemotherapy. 2012;56(1):174-8.
144. Nywening AV, Thorn HI, Xie JH, Martin-Vicente A, Guruceaga X, Ge WB, et al. Loss of the Aspergillus fumigatus spindle assembly checkpoint components, SldA or SldB, generates triazole heteroresistant conidial populations. Microbiol Spectr. 2025.
145. Schoofs A, Odds FC, Colebunders R, Ieven M, Wouters L, Goossens H. Isolation of Candida species on media with and without added fluconazole reveals high variability in relative growth susceptibility phenotypes. Antimicrobial Agents and Chemotherapy. 1997;41(8):1625-35.
146. Gautier C, Maciel EI, Ene IV. Approaches for identifying and measuring heteroresistance in azole-susceptible Candida isolates. Microbiol Spectr. 2024;12(4):20.
147. Marr KA, Lyons CN, Ha K, Rustad TR, White TC. Inducible azole resistance associated with a heterogeneous phenotype in Candida albicans. Antimicrobial Agents and Chemotherapy. 2001;45(1):52-9.
148. Claudino ALR, Peixoto RF, Melhem MSC, Szeszs MW, Lyon JP, Chavasco JK, et al. MUTANTS WITH HETERORESISTANCE TO AMPHOTERICIN B AND FLUCONAZOLE IN CANDIDA. Brazilian Journal of Microbiology. 2009;40(4):943-51.
149. Ben-Ami R, Zimmerman O, Finn T, Amit S, Novikov A, Wertheimer N, et al. Heteroresistance to Fluconazole Is a Continuously Distributed Phenotype among Candida glabrata Clinical Strains Associated with In Vivo Persistence. Mbio. 2016;7(4):12.

150. Duxbury SJN, Bates S, Beardmore RE, Gudelj I. Evolution of drug-resistant and virulent small colonies in phenotypically diverse populations of the human fungal pathogenCandida glabrata. Proc R Soc B-Biol Sci. 2020;287(1931):9.
151. Zhai B, Liao C, Jaggavarapu S, Tang YY, Rolling T, Ning YT, et al. Antifungal heteroresistance causes prophylaxis failure and facilitates breakthrough Candida parapsilosis infections. Nat Med. 2024:29.
152. Varma A, Kwon-Chung KJ. Heteroresistance of Cryptococcus gattii to Fluconazole. Antimicrobial Agents and Chemotherapy. 2010;54(6):2303-11.
153. Ferreira GF, Santos JRA, da Costa MC, de Holanda RA, Denadai AML, de Freitas GJC, et al. Heteroresistance to Itraconazole Alters the Morphology and Increases the Virulence of Cryptococcus gattii. Antimicrobial Agents and Chemotherapy. 2015;59(8):4600-9.
154. Mondon P, Petter R, Amalfitano G, Luzzati R, Concia E, Polacheck I, et al. Heteroresistance to fluconazole and voriconazole in Cryptococcus neoformans. Antimicrobial Agents and Chemotherapy. 1999;43(8):1856-61.
155. Yamazumi T, Pfaller MA, Messer SA, Houston AK, Boyken L, Hollis RJ, et al. Characterization of heteroresistance to fluconazole among clinical isolates of Cryptococcus neoformans. Journal of Clinical Microbiology. 2003;41(1):267-72.
156. Sionov E, Chang YC, Garraffo HM, Kwon-Chung KJ. Heteroresistance to Fluconazole in Cryptococcus neoformans Is Intrinsic and Associated with Virulence. Antimicrobial Agents and Chemotherapy. 2009;53(7):2804-15.
157. Liu CL, Gao QY, Li YX, Yu JH, Yu SY, Chen XF, et al. Investigation of voriconazole heteroresistance in clinical isolates of Trichosporon asahii from a multicenter study in China. Microbiol Spectr. 2025.
158. Wang ZY, Chen QW, Zhang JY, Yan HL, Chen YC, Chen C, et al. High prevalence of unstable antibiotic heteroresistance in cyanobacteria causes resistance underestimation. Water Res. 2021;202:10.
